# Interleukin 27-induced photoreceptor survival is associated with suppression of a novel Muller glia subpopulation

**DOI:** 10.1186/s12964-026-02885-1

**Published:** 2026-04-16

**Authors:** Laura D. McGee, Brandon M. Bessen, James J. Dollar, Marco Galizia, Saili Khorjekar, Ashley N. Zuniga, Stefan Kurtenbach, Abigail S. Hackam

**Affiliations:** 1 Department of Ophthalmology, Bascom Palmer Eye Institute, University of Miami Miller School of Medicine, 1638 NW 10th Ave, Miami, FL 33136 USA; 2 Sylvester Comprehensive Cancer Center, Interdisciplinary Stem Cell Institute (ISCI), University of Miami Miller School of Medicine, Miami, FL 33136 USA

**Keywords:** Retinal degeneration, rd10, Neuroprotection, Neuroinflammation, IL-27, Muller glia, snRNA-seq

## Abstract

Retinal degenerations are associated with aberrant activation of inflammation. Interleukin-27 (IL-27) is a cytokine with anti-inflammatory and neuroprotective activities in the CNS. However, the molecular and cellular mechanisms by which IL-27 mediates its neuroprotective effects remain unclear. The rd10 mouse model of retinal degeneration received an intravitreal injection of IL-27 or saline prior to degeneration. IL-27 induced sustained retina protection for at least four weeks after injection. SnRNA-seq analysis demonstrated suppression of a unique Muller glia subpopulation in IL-27 treated mice. Moreover, snRNA-seq revealed differential expression of distinct gene categories across glial and photoreceptor cell types, including genes involved in ATP synthesis, metabolism, protein translation, apoptosis, inflammation and extracellular matrix. Therefore, reducing reactive inflammation in early retinal disease with IL-27 leads to changes in numerous cellular pathways and sustained neuroprotection in rd10 mice. Additionally, our findings suggest that suppression of a specific Muller glia subpopulation contributes to the neuroprotective effect of IL-27. Therefore, this study identified new pathways of neuroprotection in the retina.

**Supplementary Information:**

The online version contains supplementary material available at 10.1186/s12964-026-02885-1.

## Key findings

1) Intravitreal injection of the IL-27 cytokine in retinal degeneration 10 (rd10) mice early in disease leads to sustained photoreceptor protection for at least four weeks.

2) IL-27 - mediated photoreceptor protection is associated with suppression of a novel Muller glia subpopulation and alteration of extracellular matrix and metabolic pathways in glia.

3) These results demonstrate that IL-27 is a potent protective molecule that alters the inflammatory and glial characteristics of the retina to induce photoreceptor survival.

**Supplementary Information:**

The online version contains supplementary material available at 10.1186/s12964-026-02885-1.

## Background

Inherited retinal diseases (IRD) belong to a large family of conditions that are characterized by retinal dysfunction and degeneration, leading to progressive vision loss and in severe instances resulting in blindness. Retinitis pigmentosa (RP) is a class of IRD with symptoms that include night blindness, peripheral vision loss and eventual deterioration of central and color vision due to rod then cone photoreceptor death [1]. While experimental therapies such as gene replacement strategies offer benefits to specific patients, treatment options are limited and there are no cures for the majority of affected individuals [2]. Moreover, because RP is associated with mutations in over 90 genes, a major focus of the field is on identifying mutation-independent approaches that halt or delay degeneration.

The well-characterized rd10 mouse model of RP is a mouse line in which rod and cone photoreceptors degenerate in a central to peripheral pattern due to a spontaneous missense mutation in the visual cycle gene Pde6β [3]. In rd10 mice, rod photoreceptor degeneration peaks at approximately post-natal day (P) 25–30 followed by secondary cone degeneration, resulting in near-total loss of photoreceptors by P45-P60 [4]. Early structural changes in the inner segment/outer segment region (IS/OS) are evident prior to photoreceptor death at P14-18 [5], and hyperreflective opacities in the vitreous that represent inflammatory cells are visible by OCT at P20 [5]. The predictable and consistent degeneration of the rd10 mouse makes it useful for investigating mechanisms of photoreceptor death and characterizing the beneficial effects of experimental IRD treatments.

Inflammatory changes during retinal degeneration have been demonstrated in multiple studies in humans and mouse IRD models. The current understanding is that inflammatory cells, primarily microglia and macrophages, contribute to the progression of retinal degeneration [6–8]. Neuroinflammatory changes in rd10 include inflammatory cell migration to the outer nuclear layer (ONL), increased expression of inflammation genes and activation of oxytosis/ferroptosis and other cell death pathways [9, 10]. Furthermore, various anti-inflammatory agents, including broad-acting drugs and targeting specific inflammatory pathways, provide short-term delay of rod and cone degeneration [7, 9, 11]. However, inflammation also confers beneficial effects in the retina, including phagocytosis of dead cells and release of protective molecules [12, 13]. Therefore, further characterization of the roles of inflammatory mediators across different stages of degeneration is essential for understanding the roles of neuroinflammation in IRD and guiding development of more effective treatments.

Interleukin-27 (IL-27) is an endogenously expressed cytokine that has anti-inflammatory functions in the CNS and pro-inflammatory effects in cancerous tissues [14, 15]. We previously demonstrated that an intravitreal injection of recombinant IL-27 improved retinal function in rd10 mice when measured one week after injection and rescued vision behavior two weeks after injection [14]. IL-27-mediated protection was associated with reduced pro-inflammatory proteins and lower expression of markers of activated microglial but not fewer microglia cells [14]. IL-27 was also shown to be neuroprotective elsewhere in the CNS, including in a murine ischemia-reperfusion stroke model [16] and intracranial hemorrhage [17]. However, the basis for IL-27 mediated anti- or pro-inflammatory pathways in different tissues is unknown, and the molecular and cellular mechanisms by which IL-27 induces its neuroprotective effects are not understood. Additionally, the duration of IL-27-mediated retinal protection after reducing inflammation at the early stages of pathology has not been tested.

In this study, we investigated the mechanisms and duration of photoreceptor rescue by IL-27 in the rd10 mouse model. We tested the hypothesis that reducing inflammation at an early stage of degeneration would prolong vision rescue. We demonstrated that IL-27 induces sustained photoreceptor protection for at least four weeks after injection. Additionally, snRNA-seq analysis demonstrated differential expression of translation, inflammation, cell survival and metabolism genes in microglia, Muller glia and photoreceptors. Furthermore, cluster analysis demonstrated suppression of a unique Muller glia subpopulation in IL-27-treated mice. Therefore, these findings demonstrate that reducing reactive inflammation prior to the peak of photoreceptor degeneration using IL-27 leads to changes in Muller glia populations and alterations in the retinal inflammatory environment that contribute to sustained neuroprotection.

## Methods

### Intravitreal injections

All animal experiments followed the ARVO statement for the Use of Animals in Ophthalmic and Vision research guidelines and were approved by the Animal Care and Use Committee at the University of Miami. The rd10 mice (B6.CXB1-*Pde6b*^*rd10*^/J, stock number 004297) were purchased from Jackson Laboratory (Bar Harbor, ME). Mice were housed in cages with standard enrichment materials and control and experimental treated mice were kept at equivalent distances from overhead lighting in a 12–12 h light-dark cycle. Mice were divided into experimental or control groups at random and investigators were masked to the identity of the treatments for all analyses. For intravitreal injections, mice at post-natal day (P) 18 were anesthetized using isoflurane and 0.5% proparacaine hydrochloride drops were applied to the corneas. The mice received intravitreal injections of one microliter of either sterile phosphate-buffered saline (vehicle control) or 20 ng recombinant carrier-free mouse IL-27 (BioLegend, San Diego, CA) using a 33-gauge Hamilton needle into one eye, with the fellow right eye remaining uninjected. The 20 ng dose was shown previously to be optimal for photoreceptor protection [18]. Only mice with successful injections without bleeding or ocular swelling were included in the study. Humane euthanasia was performed using CO_2_ at various timepoints after injection and the eyes were immediately removed for retina dissection or were processed for cryosectioning.

### Optomotor response tracking (OMR)

OMR was used to measure visual acuity in mice at various timepoints (P25, 45 and 60) using an optometry device (Cerebral Mechanics, Inc.), following the procedures described in [18, 19]. The mice were placed on a circular platform in the center of the observation chamber surrounded by four monitors and a camera above the platform. Reflexive head tracking movements in response to rotating black and white columns were determined by at least two investigators masked to the treatments. Stepwise decreases in column thickness were used to identify the visual acuity threshold. Head tracking to the right in response to a clockwise rotating pattern of columns measured visual acuity of the left eye, while tracking to the left in response to a counterclockwise pattern measured visual acuity of the right eye. We compared only the responses of the left eyes (injected with IL-27 or saline, or uninjected for the untreated mice). Samples sizes, representing individual mice, are: P25: UT *n* = 6, saline *n* = 14, IL-27 *n* = 14; P45: UT *n* = 16, saline *n* = 7, IL-27 *n* = 9; P60: UT *n* = 18, saline *n* = 6, IL-27 *n* = 5. Animals were removed from the study for tissue or molecular analysis at each timepoint.

### Electroretinogram (ERG)

Light-evoked retinal responses were measured using the LKC UTAS BigShot ERG apparatus with a Ganzfeld dome, as described previously [20]. Mice at age P45 were anesthetized with an intraperitoneal injection of a ketamine/xylazine mixture and dilation drops were placed on both eyes. Platinum wire corneal electrodes were placed on each eye, a ground electrode was inserted adjacent to the base of the tail and a reference electrode was inserted subcutaneously in the forehead. ERG scotopic and photopic responses were recorded using ten flashes of increasing intensities of white (to measure rod-driven responses) or green (cone-driven responses) light, respectively, using a five second inter-flash interval. The response averages were determined using UTAS software and analyzed in Excel to obtain a and b wave amplitudes for each flash intensity. Because a-wave responses are absent at the P45 timepoint, b-wave responses were used instead as an indirect measure of photoreceptor health. Mouse a- and b-waves are highly correlated in normal and degenerating retinas [20]. Samples sizes, representing individual mice, are IL-27 *n* = 5, saline *n* = 8.

### Photoreceptor death

The DeadEnd Fluorometric TUNEL system kit (Promega, catalog G3250) was used to quantify dying cells on ten-micron frozen retinal sections according to the manufacturer’s instructions. Briefly, retinal sections were prepared from mice at P45 and placed on glass slides, *n* = 6 per group. The slides were washed in PBS, treated with proteinase K then incubated with TdT/nucleotide mixture containing fluorescein-12-dUTP, followed by reaction termination in 2x SSC and additional PBS washes. The slides were covered with Vectashield antifade mounting medium with DAPI and viewed with a Zeiss fluorescent microscope. TUNEL-positive and DAPI-positive cells in the ONL were counted along the retina by two independent investigators masked to the treatment identity. TUNEL counts were averaged from at least 6 non-adjacent sections per eye.

### Quantification of cone photoreceptors

Retinal cryosections from mice age P45, *n* = 4 per group, were washed in PBS three times then blocked using freshly prepared blocking solution (10% goat serum, 3% Triton X-100 in PBS) and incubated in a humidity chamber at room temperature for 30 min. The slides were then treated with rhodamine-labeled peanut agglutinin (PNA, 5 µg/mL, Vector Laboratories, RL-1072-5) for one hour at room temperature, washed in PBS, and then briefly air dried. The retinal sections were covered with Vectashield plus antifade mounting medium with DAPI (Vector Laboratories, H-2000), coverslips were applied, and the slides were viewed within an hour using a Carl Zeiss fluorescence microscope. Imaging was performed using DAPI to visualize nuclei and the rhodamine channel to visualize cone photoreceptor outer and inner segments. Microscope imaging parameters were kept constant for IL-27 and saline-injected mice. Cone quantification was performed in the peripheral retina of each section by tracing a 300-micron spline curve from the ciliary body along the retinal sections. A signal was considered a cone if it clearly connected to ONL and was at least 5 microns in length. Adjacent signals were counted as individual cones only if distinct projections above the ONL were visualized.

### Stat1 activity

Retinas were obtained 7 days after IL-27 or saline injection at P25 (*n* = 4 per group) and lysed in the presence of protease and phosphatase inhibitors in a volume of 100 µl. The levels of phosphorylated Stat1 (Ser727) and total Stat1 in 10 µl of retina lysate were measured using the Stat1 + total Stat1 ELISA kit (Abcam, catalog ab126455). Samples were processed according to the manufacturer’s procedures and Stat1 levels were measured in duplicate for each retina using a BMG LabTech Omega plate reader at 450 nm and compared to the standard curve. The ratio of phospho-Stat1 to total Stat1 was calculated using absolute non-normalized values for each sample to determine activated Stat1 levels.

### Retinal cytokine immunoassay

A LegendPlex custom mouse immunoassay panel (catalog 900008466, BioLegend, San Diego, CA) was used to quantify levels of 14 inflammatory proteins in retina lysates. Retinas were isolated from IL-27 and saline-injected mice one week after injection at age P25 (*n* = 4 per group) and lysed in 100 µl of PBS/0.1% NP-40 with protease inhibitors, as described previously [7]. Samples (25 µl of retina lysate) were assayed in duplicate according to the manufacturer’s instructions, and protein levels in the standards and samples were measured using a CytoFlex S (Beckman Coulter). Protein amounts using non-normalized values were derived from standard curves generated using Legendplex data analysis software.

### Microglia cell line expression analysis

The BV2 mouse microglia cell line [21] was cultured in DMEM media with penicillin-streptomycin and FBS and treated with 300 ng/ml IL-27 or saline for 24 h. The cells were then harvested by scraping and RNA was extracted using Trizol reagent (Invitrogen) following the manufacturer’s procedures. OligodT primers were used to prepare cDNA from one microgram purified RNA using Superscript VI (Thermo Fisher) and QPCR was performed using 2X Universal SYBR Green Fast qPCR Mix (Abclonal) in an Eppendorf Realplex Mastercycler. Primers to the genes of interest (Supplemental Table 1) were designed using Primer-BLAST and were separated by at least one intron. KiCqStart primers (Millipore Sigma, KSPQ12012G) were used for *Ocm* gene amplification. Relative expression levels of the target genes were calculated using the delta-delta Ct method described in https://toptipbio.com/delta-delta-ct-pcr with the ARP gene as a reference housekeeping control gene [22]. Sample size, representing independently treated plates of cells, was *n* = 3 per group.

### Immunohistochemistry

Enucleated globes from ages P20 and 25 (*n* = 3 per group) were fixed in 4% paraformaldehyde, washed with PBS and transferred to a 5% sucrose solution with constant mixing overnight at 4 °C. The eyes were then transferred to increasing concentrations of sucrose solution (10–20%) then embedded in optimal cutting temperature compound solution (Tissue-Tek) and flash frozen. The embedded eyes were sectioned into ten-micrometer sections and mounted onto glass slides. For immunostaining, slides were washed with PBS for 5 min, incubated in blocking buffer (10% goat serum in 0.3% Triton X-100/PBS) then incubated with primary antibodies diluted 1:200 to 1:400 in 2% goat serum/0.3% Triton X-100/PBS solution overnight at 4 °C (antibodies used in this study are listed in Table 1). After three consecutive washes in PBS, secondary antibody solution was added for 30 min at room temperature followed by additional PBS washes. The slides were briefly dried and then counterstained with DAPI in Vectashield mounting media. Retina sections were imaged with a Zeiss fluorescent microscope using equivalent microscope settings among treatment and control groups. Negative controls omitted the primary antibody.

**Table 1:** Antibodies used in the study

Antibody	Company	RRID	Catalog #
Agmo (TMEM195)	Proteintech	AB_2878845	21355-1-AP
Dio2	Proteintech	AB_2880538	26513-1-AP
Fbln1	Proteintech	AB_10859100	20425-1-AP
Frmpd4	Proteintech	AB_2879369	23943-1-AP
Glis1	Proteintech	AB_2879216	23138-1-AP
Lsamp	Proteintech	AB_10597712	13600-1-AP
Ninj2	Proteintech	AB_10642559	14085-1-AP
Nostrin	Proteintech	AB_10666167	20116-1-AP
Nox4	Proteintech	AB_10638146	14347-1-AP
Pou3f2 (BRN2)	Proteintech	AB_2167371	14596-1-AP
Rorb	Abcam	-	ab314650
Tox2	Proteintech	AB_2878821	21162-1-AP
anti-Rabbit AF 488	Thermo Fisher Scientific	AB_2576217	A11034

### Single nuclei RNA sequencing and integration

Single-nucleus RNA sequencing was performed by Novogene Inc. (Sacramento, CA). Nuclei were isolated from frozen mouse retinas that were collected two days after injection (age P20, *n* = 3 per group), using the Chromium Nuclei Isolation Kit (10x Genomics). The snRNA-seq libraries were prepared with the Chromium Single Nuclei 3′ Library & Gel Bead Kit (10x Genomics) and sequenced on the Illumina NovaSeq X platform. FASTQ files from single-nucleus RNA-seq were processed with Cell Ranger (10x Genomics, v9.0.1) against the 10x Genomics mouse reference. The filtered matrices were processed in Seurat (v5.2.1); nuclei were retained if nUMI > 500, nFeature_RNA ≥ 200, and percent.mt < 5%. Doublets were identified with DoubletFinder (v2.0.4) using an expected doublet rate of 8% per sample with homotypic adjustment, and predicted doublets were removed prior to downstream integration.

Sample integration was conducted with Harmony-based (v1.2.3) methodology implemented via Seurat using the first 25 dimensions. To cluster single nuclei, the nearest neighbor graph was constructed and used for Louvain cluster identification with the Seurat functions, *FindNeighbors* and *FindClusters*, respectively. UMAP dimensionality reduction was generated with the Seurat function *RunUMAP*. Cell cycle scoring was conducted with the Seurat function, *CellCycleScoring*, using reference “G2M” (*n* = 54 genes) and “S” phase (*n* = 43 genes) genes from converted to mouse orthologs. Additionally, clustering of Muller cell subpopulations was conducted by scVI integration via *scvi-tools* (v1.0.4), conducting batch correction by training with 400 epochs. Subsequent UMAP dimensionality reduction and Louvain clustering was conducted with Seurat as described with the first 10 principal components and 4000 variable features. Visualization of single-nucleus data was generated with plotting functions from Seurat, scCustomize (v3.0.1), SeuratExtend (v1.1.2), and dittoSeq (v1.18.0), with additional functions used by R packages, ggsci (v3.2.0) and cowplot (v1.1.3).

### Processing of mouse retina reference atlas and reference-based annotation of single-nucleus cohort

Reference-based annotation of snRNA-seq integrated object was conducted with a comprehensive mouse retina single-cell RNA sequencing atlas [23]. The single-cell mouse retina reference was downsampled to a maximum of 2000 cells for each cell type, and gene ensemble ID conversion to Mouse Genome Informatics gene symbols was conducted using the *M. musculus* datasets from biomart R package (v2.62.1). The reference then underwent SCTransform-based normalization with 3,000 variable features with Seurat. Following processing of reference atlas, transfer anchors between the mouse single-cell retina reference and single-nucleus cohort were calculated using log normalization and 30 dimensions with the *FindTransferAnchors* function and subsequently used to label single nuclei with the *TransferData* function from Seurat. Cell labels were verified by marker expression.

### Pseudobulk analysis of single-nucleus cohort

Following nuclei annotation, RNA counts were processed as an *SingleCellExperiment* object (v1.28.1) and used for nucleus-level quality metrics computation with the *perCellQCMetrics* function from scuttle (v1.16.0). Outlier nuclei were determined with the scuttle *isOutlier* function specifying outliers as nuclei with log2-transformed counts more than 2 median absolute deviations from the log2-transformed median count. Genes expressed by 10 or fewer nuclei were removed before library size factors were computed and log-transformed normalization of RNA counts generated using scuttle functions *computeLibraryFactors* and *logNormCounts*, respectively. Normalized count matrices for pseudobulk samples were generated by sum-based aggregation by samples and cell types. Negative binomial differential expression analysis was conducted using the DESeq2 R package (v1.46.0) with p-values adjusted by Benjamini-Hochberg procedure and with an alpha set to 0.05. Shrinkage of log2 fold changes was conducted with the *lfcShrink* function from DESeq2, using the adaptive Student’s t prior shrinkage estimator implemented with the R package, apeglm (v1.28.0). Principal component analysis (PCA) of pseudobulk samples was conducted with sum-aggregated RNA counts of the top variable genes. Variable genes which were determined with the *rawVars* function from matrixStats (v1.5.0) from regularized log transformed counts generated with the *DESeqDataSetFromMatrix* and *rlog* functions from DESeq2. PCA plots were generated with the DESeq2 function, *plotPCA*. Visualization of differential expression analysis was conducted with the ggplot2 (v3.5.1) and EnhancedVolcano R packages (v1.24.0).

### Single-nucleus differential expression analysis and gene set enrichment analysis (GSEA)

The Seurat function *FindAllMarkers* with Wilcoxon testing was used for comparison of multiple cell clusters for markers. For differential expression analysis of snRNA-seq counts were analyzed with the R package, edgeR (v4.4.2), using QLF-based methodology and setting cellular detection rate as a covariate [24, 25]. The percentage of cells expressing a gene per group was computed with the function *Percent_Expressing* from scCustomize. False discovery rate (FDR) correction was used to adjust p-values. GSEA was conducted using clusterProfiler (v4.14.6) with significantly differentially expressed genes (adjusted p-value < 0.05) ranked according to log_2_ fold change. Reference mouse gene set collections were accessed with the R package, msigdbr (v7.5.1). Additional functions and visualization tools implemented from DOSE (v4.0.0), enrichplot (v1.26.6), ggplot2, and EnhancedVolcano R packages.

### Cell-cell communication network analysis

The R package CellChat (v2.1.2) was used to infer the cell-cell communication network, signaling strengths and interaction counts for cell populations. The murine CellChat ligand-receptor database was used for network construction. Cell-cell communication networks were analyzed by individual samples and by treatment group for IL-27 treated and saline control retinas. Log-normalized RNA expression was used to generate the CellChat object, which was subsequently processed by CellChat functions, *identifyOverExpressedGenes* and *identifyOverExpressedInteractions*. The communication probabilities and inferred communication network was generated with *computeCommunProb*. Cell-cell communication was filtered for signal present in at least 10 nuclei with *filterCommunication*. Pathway-level summation of ligand-receptors signal within cell-cell communication networks was calculated with *computeCommunProbPathway* and aggregated with *aggregateNet*. Network centrality scores were calculated by *netAnalysis_computeCentrality* function. Signaling network functional similarity was calculated with *computeNetSimilarity* and used for Manifold-based classification with the functions, *netEmbedding* and *netClustering*, respectively. The function, *rankNet*, was utilized to compute ranked signaling weight differences between treatment groups. Incoming and outgoing signaling for cell types were compared and plotted with the function, *netAnalysis_signalingChanges_scatter*. Significance was determined by two-tailed Wilcoxon test.

### Gene ontology enrichment analysis

Differentially expressed genes from the snRNA-seq data with FDR < 0.05 were categorized using Gene ontology (GO) terms with PANTHER software, Version 19.0 [26]. Gene enrichment was determined using *Mus musculus* as the reference list and Fisher’s exact test for the following terms: Biological process, Molecular function and Protein class. Significant fold enrichment (positive or negative) for the various GO categories was indicated by FDR < 0.05. Primary literature sources were used to confirm biological processes and functional annotations when necessary.

### Statistical analysis

GraphPad Prism was used for plotting data and statistical analyses. Statistical analysis of OMR tests used two-way ANOVA followed by Tukey’s multiple comparison tests. One-way ANOVA was used for analysis of photoreceptor rows, and Student’s t-test was used for comparisons between two groups for QPCR, Stat1 activation, cone photoreceptors and immunoassays. Comparisons of the total number of TUNEL-positive cells used weighted means and combined variances from IL-27 and saline treatments calculated at P25 [18] and P45, followed by analysis using unpaired t-test with Welch’s correction. ERGs were analyzed using a Wilcoxon rank sum test.

## Results

### IL-27 alters the inflammatory environment in the retina

**Fig. 1:**
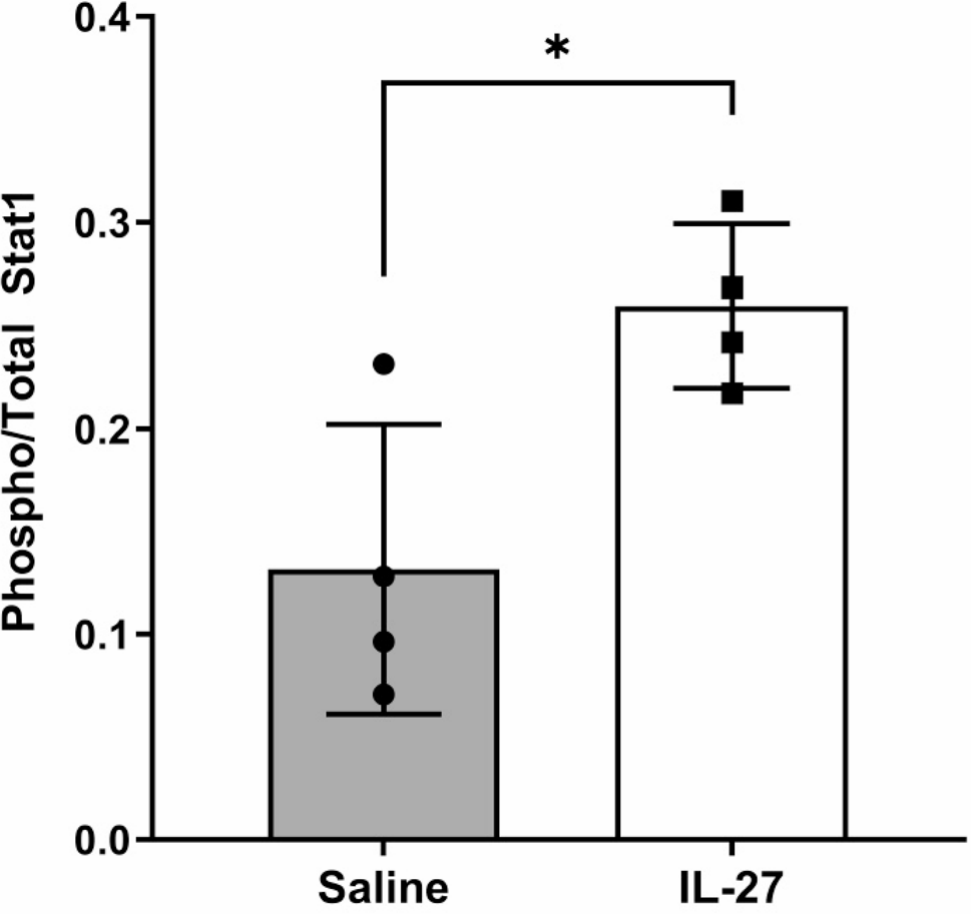
IL-27 induces Stat-1 activation one week after injection. ELISA assays on retina lysates measured total and activated (phospho-Ser727-STAT1) levels at P25. The ratio of phospho-Stat1 to total Stat1 is shown for retinas from IL-27 injected eyes and saline control. *p=0.0196, n=4/group

IL-27 regulates inflammatory signaling pathways by activating Jak-Stat pathways, primarily Stat1 and Stat3, in inflammatory cells and various tissues, including retina [27, 28]. Stat1 is an important transcriptional regulator of numerous pro- and anti-inflammatory genes and an effector of normal retina function [29]. To determine whether IL-27 signaling remains active one week after injection, the amount of Stat1 activation (phospho STAT1/total STAT1) was measured by ELISA assays in retinal lysates after injection of the optimal IL-27 dose established previously [18]. As shown in Fig. 1, Stat1 activation was significantly higher in IL-27-injected eyes compared to saline-injected eyes. Although the half-life of IL-27 in the mouse eye is expected to be only ~ 1 h, as shown for related cytokines [30], this result demonstrates that Stat1 activation remains elevated one week after injection and could continue to regulate inflammatory and other cellular pathways stimulated by the initial IL-27 injection.

**Fig. 2:**
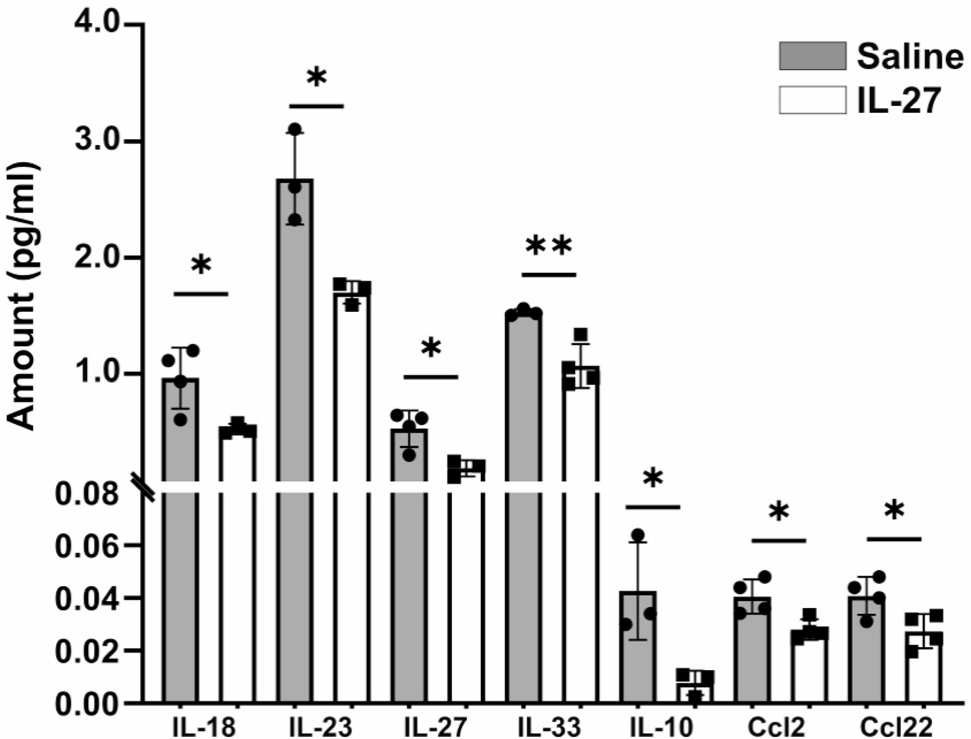
IL-27 suppresses numerous cytokines one week after injection. Multiplex immunoassays were used to measure cytokine levels in retinal lysates from mice injected with IL-27 or saline. IL-27 reduced expression of multiple pro-inflammatory cytokines. *p<0.05, **p<0.01, n=4/group

We next investigated inflammatory mediators regulated by IL-27. In a previous study, we demonstrated that intravitreal injections of IL-27 altered cytokines in the retina when measured three days after injection [18]. To determine whether IL-27-induced inflammatory changes persisted over a longer duration, particularly during the period of maximal photoreceptor degeneration, we examined levels of inflammatory proteins in retinal lysates one week after IL-27 injection using multiplex immunoassays. As shown in Fig. 2, IL-27 treatment led to reduced expression of multiple cytokines, including IL-18, IL-23, IL-27, IL-33, IL-10, CCL2, and CCL22. Several of these cytokines have pro-inflammatory functions, such as IL-18 and Ccl2, whereas others, such as Ccl22 and IL-10, function as both pro- and anti- inflammatory cytokines depending on the tissue context [31]. Interestingly, IL-27 reduces its own expression, suggesting a negative-feedback effect or overall general suppression of inflammatory pathways. Other cytokines that were examined, CxCL13, CxCL10, CCL17, IL-1β and IL-4, were not changed by IL-27 at the timepoint tested (results not shown).

**Fig. 3:**
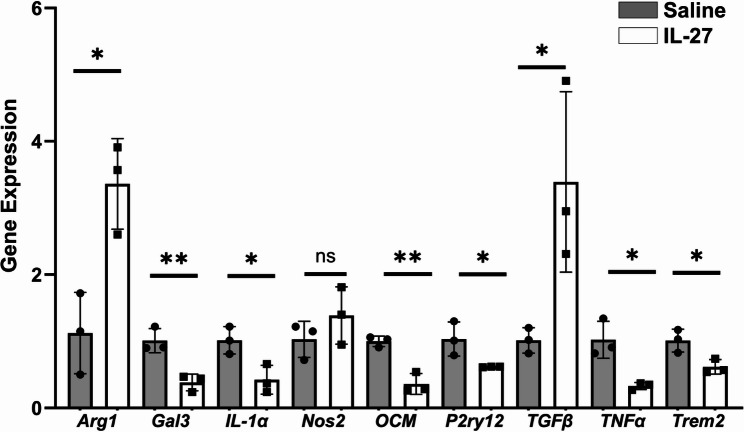
IL-27 regulates inflammatory genes in a microglia cell line. Cultured mouse microglia BV-2 cells were treated with IL-27 or saline and analyzed by qPCR. Increased expression of anti-inflammatory Arg1 and TGFβ and reduced expression of multiple pro-inflammatory cytokines confirm regulation of inflammation by IL-27. *p<0.05, **p<0.01, n=3/group

The microglia cell line BV2 expresses IL-27 and IL-27 receptor genes [18]. To determine the effect of IL-27 on microglial expression of inflammatory genes, BV-2 cells were treated with IL-27 or saline vehicle control and gene expression was examined by qPCR. As shown in Fig. 3, IL-27 reduced expression of multiple pro-inflammatory genes including Trem2, P2ry12, TNFα, OCM, Gal3 and IL-1α. Additionally, IL-27 increased expression of the anti-inflammatory cytokine TGFβ and Arg1, a marker of anti-inflammatory microglia, whereas NOS2, a marker of pro-inflammatory microglia, was not changed. Therefore, IL-27 regulates inflammatory gene expression in microglia, which may contribute to changes in the overall inflammatory microenvironment in the retina.

### IL-27 induces sustained photoreceptor survival

**Fig. 4:**
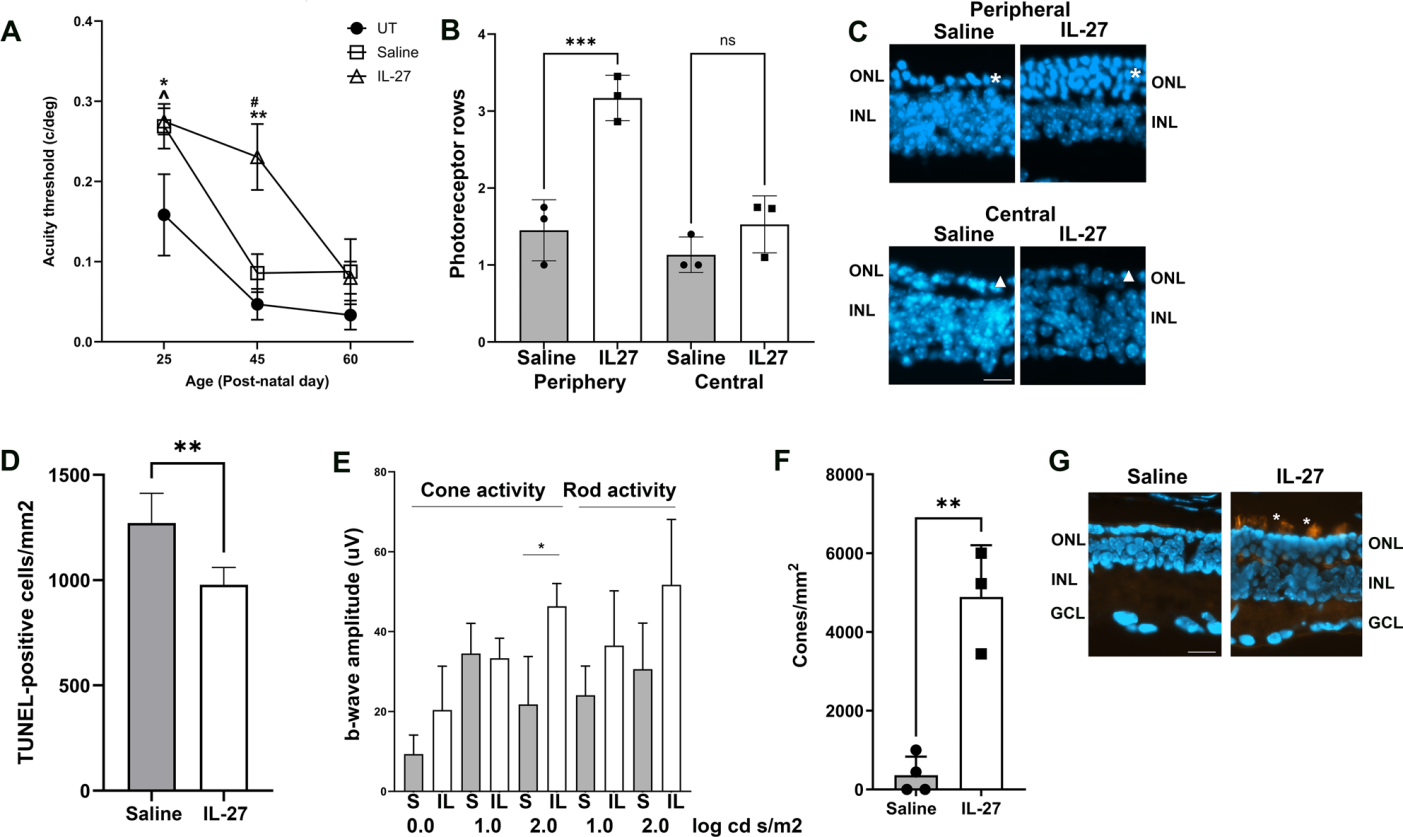
Delivery of IL-27 early in disease leads to sustained photoreceptor rescue measured four weeks after intravitreal injection. Mice were injected at P18 and phenotypes were analyzed at P45. **A** IL-27 increases visual acuity at P45 compared to saline and untreated (UT) rd10 mice, indicating a delay in vision loss following injection. Only the responses of the left eyes are shown (injected with IL-27 or saline, or uninjected for the untreated mice). **p=0.0003, IL-27 vs. UT; #*p*=0.0121, IL-27 vs. saline; *p=0.039, saline vs. UT; ^*p*=0.0192, IL-27 vs. UT. Sample sizes: P25: UT *n*=6, saline *n*=14, IL-27 *n*=14; P45: UT *n*=16, saline *n*=7, IL-27 *n*=9; P60: UT *n*=18, saline *n*=6, IL-27 *n*=5. **B** IL-27 injection resulted in more photoreceptor rows in the peripheral retina compared to saline injection controls at P45, indicating photoreceptor rescue. *p=0.001, *n*=3/groups. There was no difference in the number of photoreceptor rows between treatments in the central retina. **C** Representative images showing more photoreceptor nuclei in the outer retina in IL-27 injected eyes compared with saline controls in the peripheral (asterisks) but not the central retina (triangles). The DAPI signal brightness was increased for the central retina images while constructing the figure to better visualize the retinal layers. **D** IL-27 injection reduced the total number of dying photoreceptors in the ONL, measured by TUNEL assay as in [18], compared to saline *p=0.022, *n*=6 per group. **E** ERG analysis on IL-27 injected mice showed higher cone-driven responses than saline controls at the highest flash intensity at P45 but not other intensities, indicating a mild but significant improvement in visual function. Scotopic (white light, rod responses) and photopic (green light, cone responses) b-wave amplitudes for different flash intensities in log cd.s/m2 are shown. *p=0.045, averages and standard error are shown, IL-27 (IL) *n*=5, saline (S) *n*=8. **F** PNA-labeling to visualize cone photoreceptors shows higher numbers of cones in IL-27 injected eyes at P45 compared to saline *p=0.013, *n*=4 per group. **G** Representative images of PNA-labeled cone photoreceptors are shown (orange, asterisks). Scale bars, 25 µm. ONL, outer nuclear layer, INL, inner nuclear layer, GCL, ganglion cell layer

We next used several approaches to characterize the effect of IL-27 on retinal structure and function to determine whether inflammatory changes lead to sustained retinal protection 4 and 6 weeks after injection. Visual acuity was measured using the optomotor response over time across different treatment groups. As shown in Fig. 4A, visual acuity measured one week after injection at P25 was equivalent between saline and IL-27 injected groups despite higher ERG recordings in IL-27 injected mice at this timepoint measured previously [18]. Both treatments were ~ 70% higher than untreated rd10 mice at P25. When acuity was measured in the same animals at four weeks post-injection (P45), the IL-27-injected mice maintained visual acuity and it was almost three-fold higher in the IL-27 injected mice than saline-injected mice, indicating significant retinal protection by IL-27. In contrast, the visual acuity of the saline injected group was equivalent to untreated mice at P45 (Fig. 4A). The transient protective effect of saline at P25 was likely due to injury-induced neurotropin expression [32]. When tested 6 weeks after injection (P60), visual acuity was reduced in the IL-27 group and all three groups showed equivalent acuity. Therefore, IL-27 injection early in degeneration preserved vision for at least four weeks, delaying the natural decline in vision in this IRD model.

To assess photoreceptor survival, rows of nuclei in the ONL were counted from retinas obtained four weeks after injection. At this timepoint in rd10 mice, the ONL nuclei are expected to be a single row representing mostly cone photoreceptors [4]. As shown in Fig. 4B-C, the number of photoreceptor rows in the peripheral retina of IL-27-injected mice was 3.17 *±* 0.295, which was approximately two-fold higher than photoreceptor rows in saline-injected mice (1.45 *±* 0.397, *p* = 0.0012). The number of photoreceptor rows in the central retina was equivalent between the treatment groups (IL-27: 1.15 *±* 0.215, saline: 1.57 *±* 0.419, *p* = 0.4702), as expected due to more severe degeneration in the central retina. Consistent with increased photoreceptor row counts in IL-27 injected mice, TUNEL assays demonstrated that the total number of TUNEL-positive photoreceptors was lower in retinas from IL-27-injected mice compared to saline-injected mice (IL-27: 978 cells/mm^2^ *±* 141, saline: 1271 *±* 82 cells/mm^2^, *p* = 0.0022), confirming lower photoreceptor death in IL-27-injected retinas (Fig. 4D).

ERG analysis was performed on the mice four weeks after injection at P45 to quantify whether IL-27 rescues functional responses to light. ERG responses in rd10 mice are usually minimal to absent at P45 [33]. Although ERG amplitudes were low as expected, cone-driven b-wave amplitudes in IL-27-injected mice were two-fold higher than those in saline-injected mice at the highest light intensity level, although they were not significantly different at lower light intensities (Fig. 4E). Rod-driven b-waves (Fig. 4E), and rod and cone a-waves (data not shown) were minimal and equivalent in both groups. To determine whether higher photopic ERGs in IL-27-injected mice corresponded to an increase in the number of surviving cone photoreceptors, we used PNA staining to detect and quantify cones. IL-27 injected eyes showed extensive PNA labeling and we identified numerous cone photoreceptors in the outer retina, although the cone outer segments had reduced lengths. In contrast, saline injected retinas had minimal PNA labeling, and all detected cones had rudimentary and extremely dysmorphic outer segments. Quantification of cones in the peripheral retina demonstrated more cones in IL-27 injected eyes (IL-27: 4,889 *±* 1,311 cones/mm^2^, saline: 360 *±* 474, *p* = 0.0013) (Fig. 4F-G). The central retina had no discernible cone photoreceptors in either IL-27 and saline injected retinas (data not shown), consistent with lower photoreceptor nuclei counts in the central region (Fig. 4B-C). Together, the findings of higher visual acuity, increased photoreceptor counts, reduced TUNEL-positive cells and increased ERG responses in the IL-27-injected mice indicate partial rescue of photoreceptor function that persisted for 4 weeks after the initial injection.

### Integration and annotation of mouse retina single nuclei

**Fig. 5:**
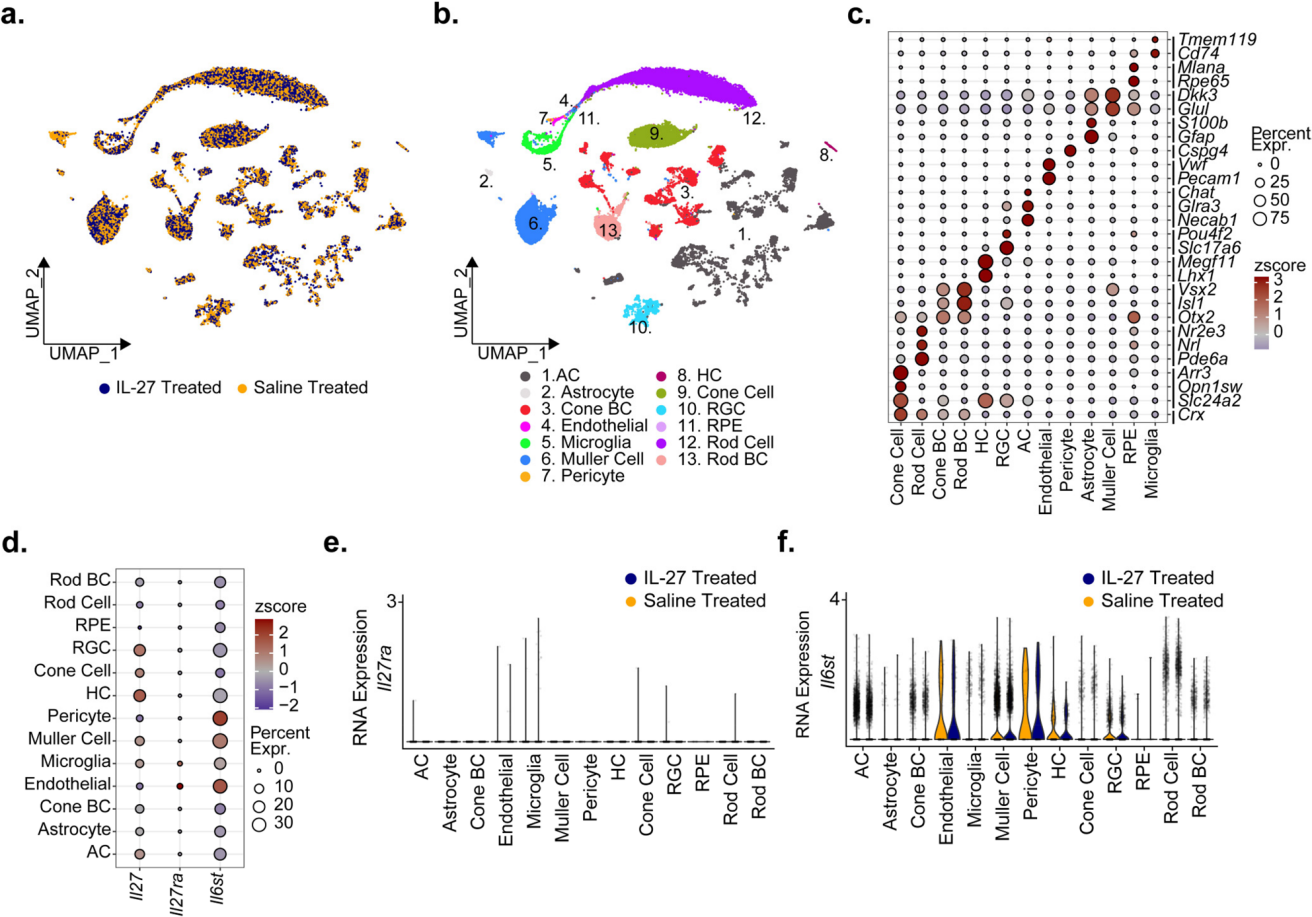
Overview of single-nucleus RNA sequencing (snRNA-seq) to deconvolute IL-27 treatment response in murine retina at single cell resolution. (**a-b**) Uniform Manifold Approximation and Projection (UMAP) dimension reduction plot displaying 51,988 single nuclei from IL-27 treated (n=3) and saline control treated (n=3) murine retina. Cells annotated by (**a**) treatment cohort and (**b**) major cell type. (**c**) Verification of cell type annotation by dot plot displaying the RNA expression of cell type associated genes by major populations. Dot color indicates the zscore (scaled average expression) while the size indicates the percentage of cells expressing that gene. (**d**) RNA expression of Il27 and IL-27 receptors, Il27ra and Il6st, in all retina cell types exhibited by dot plot. (**e-f**) RNA expression of IL-27 receptors, (**e**) Il27ra and (**f**) Il6st, in all cell type populations split by treatment group demonstrated with stacked violin plot. Abbreviations: Expr., Expressed. BC, bipolar cells; HC, horizontal cells; RGC, retinal ganglion cells; AC, amacrine cells; RPE, retinal pigmented epithelial cells

Analysis of Stat1 and cytokines at P25 indicated changes in inflammatory pathways lasted at least one week after injection. To investigate earlier molecular and cellular changes in the retina in response to IL-27 that may contribute to photoreceptor protection at later timepoints, we performed snRNA-seq on retinas obtained at P20, two days after IL-27 or saline injection. This timepoint was chosen because cells in the ONL are actively dying but cell loss is minimal, and it is at least five days before the peak of degeneration at P25-P30 when dramatic thinning of the ONL is observed [4]. Following quality filtering and doublet removal, we performed Harmony-based integration, dimensionality reduction, and clustering of 51,988 single nuclei from treated mouse retina (Supplemental Fig. 1a), yielding similar numbers of captured nuclei for both IL-27 (*n* = 25,934 nuclei) and saline (*n* = 26,054 nuclei) treatment cohorts (Fig. 5A). Next, we processed a comprehensive single-cell mouse retina atlas as a reference [23] to conduct reference-based annotation of our single nuclei cohort. This annotation revealed clusters of retinal cell populations (Fig. 5B) and subpopulations (Supplemental Fig. 1b) exhibiting strong overlap with unsupervised Louvain clusters (Supplemental Fig. 1c). Annotation of cell type was verified by expression of associated markers for identified cell types (Fig. 5C), confirming the presence of bipolar cells (BC), horizontal cells (HC), retinal ganglion cells (RGC), amacrine cells (AC), rod and cone photoreceptors, Muller glia, microglia and retinal pigmented epithelial cells (RPE).

Once annotated, we assessed the expression of *Il27* and its associated receptors, *Il27ra* and *Il6st*, in the retinal cell populations (Fig. 5D). *Il27* expression appeared in several cell populations, including neurons (RGC, HC) and glia, consistent with previous IHC analysis [18]. In contrast, *Il27ra* was expressed by endothelial cells and microglia but was not widely detected across retinal cell types. The *Il6st* receptor was expressed in several cell types, with pericytes, endothelial cells and Muller cells appearing to be some of the strongest expressors of *Il6st* (Fig. 5D). To assess whether transcription of these receptors was altered with IL-27 treatment, we conducted differential expression analysis for each cell population comparing IL-27 treated versus saline control retinas. Single-nucleus differential expression analysis revealed that neither *Il27ra* nor *Il6st* were significantly dysregulated with IL-27 treatment for any cell type (Fig. 5D-F).

Next, gene annotation was used to identify cellular pathways regulated by IL-27. Differentially expressed genes in each cell type with FDR < 0.05 were analyzed for enrichment of molecular functions and biological processes using the PANTHER software tool. The GO categories (italicized henceforth) of upregulated genes in IL-27 treated eyes showed unique cellular pathways and protein classes in the various cell types (Table 2). For example, upregulated genes in rod photoreceptors showed over-representation of genes involved in normal rod function (*photoreceptor cell maintenance*, 10-fold enriched, *synaptic vesicle clustering*, 23-fold), consistent with IL-27 protecting photoreceptors from damage and degeneration. In cones, enriched GO categories included *cGMP metabolic process* (29-fold enriched) and *neural retina development* (5-fold enriched), and in Muller glia, *phosphodiesterases* and *TORC2 signaling* were enriched (5- and 16-fold, respectively), indicating increased signaling activity in IL-27 injected eyes. Muller glia also included *defense response to virus* (6-fold) in the upregulated gene list, indicating regulation of inflammatory signaling. Upregulated genes in microglia, the predicted direct target cells of IL-27, included *positive regulation of cell communication* (4-fold enriched), *cellular response to oxygen-containing compound* (5-fold) and several tissue development categories.

**Table 2:** Top GO categories for Muller glia, rods, cones and microglia by snRNA-seq from Panther bioinformatics analysis

Cell Type	GO Category	# Genes	Fold Enrichment	Raw P value	FDR
Microglia	**Increased**				
	Positive regulation of response to stimulus	14	3.4	2.20E-05	2.80E-02
	Forebrain neuron differentiation	4	26.52	1.54E-05	2.93E-02
	Cellular response to oxygen-containing compound	10	4.91	2.19E-05	3.03E-02
	Positive regulation of cell communication	12	3.67	5.02E-05	3.83E-02
	**Decreased**				
	Cytoplasmic translation	10	29.77	1.20E-12	1.83E-08
	Visual phototransduction	6	26.59	9.95E-06	9.64E-02
	Oxidative phosphorylation	6	19.83	5.71E-07	1.24E-03
	Regulation of intrinsic apoptotic signaling pathway	6	12.06	1.02E-05	1.20E-02
Muller Glia	**Increased**				
	Phosphodiesterase	9	5.39	3.42E-05	1.12E-03
	Defense response to virus	8	5.66	6.55E-05	3.16E-03
	TORC2 signaling	3	16.54	4.50E-04	1.68E-02
	Non-receptor serine/threonine protein kinase	25	1.92	2.34E-03	4.61E-02
	**Decreased**				
	Translation at postsynapse	45	14.93	5.81E-51	4.43E-47
	ATP synthase	14	4.74	6.66E-07	1.46E-05
	Extracellular matrix structural protein	14	3.14	1.14E-04	1.72E-03
	Oxidase	12	2.73	1.28E-03	1.48E-02
Rods	**Increased**				
	Photoreceptor cell maintenance	8	10.47	8.16E-07	4.98E-04
	Retinal rod cell differentiation	5	12.3	4.49E-05	1.14E-02
	Response to light stimulus	16	2.8	2.23E-04	3.64E-02
	Positive regulation of synaptic vesicle clustering	3	23.07	2.25E-04	3.64E-02
	**Decreased**				
	Cytoplasmic translation	22	8.49	1.40E-14	2.13E-10
	Chaperone	15	3.73	1.40E-05	6.91E-04
	Apoptotic process	47	2.23	3.29E-07	1.07E-04
	Autophagosome membrane docking	3	18.91	4.03E-04	2.82E-02
Cones	**Increased**				
	Neural retina development	8	5.29	1.38E-04	2.60E-02
	cAMP metabolic process	3	28.91	1.47E-04	3.45E-02
	Negative regulation of Hippo signaling	3	27.65	1.69E-04	3.83E-02
	Regulation of neuron migration	4	13.46	2.25E-04	4.63E-02
	**Decreased**				
	Hsp90 family chaperone	3	150	5.09E-07	1.00E-04
	Regulation of necroptotic cell death	3	21.86	3.24E-04	2.21E-02
	Regulation of apoptotic process	24	2.93	1.50E-06	2.29E-02
	Hsp70 family chaperone	3	45.4	3.52E-05	3.47E-03

Cellular pathways in the downregulated gene categories in IL-27 injected mice were shared across multiple cell types. Notably, stress response genes in the *hsp70* and *hsp90* families were prominent in the down-regulated gene list of rod and cone photoreceptors after IL-27 injection, suggesting that retinal stress is dampened by IL-27, which is consistent with its neuroprotective effect at later timepoints. Furthermore, genes involved in *protein translation* were enriched in the downregulated gene group in rods, cones, Muller glia and microglia. Similarly, genes involved in metabolism (*ATP synthase*,* oxidative phosphorylation*) were over-represented in the downregulated gene list in Muller glia and microglia. Together, these findings suggest that reduction of metabolic demand is associated with dampening neuroinflammation early in degeneration.

We next examined whether snRNA-seq analysis detected differential expression of cell survival genes. The GO categories *regulation of apoptotic process* (3-fold) and *regulation of necroptotic cell death* (22-fold) were enriched in the list of down-regulated genes in cones, consistent with reduced cellular stress. Although cones and rods show early pathologic changes at this timepoint in rd10 mice, excessive cell death is not observed until later [4]. The identification of down-regulated cell death genes in the IL-27 injected eyes suggests that early changes in cell survival genes induced by IL-27 may facilitate a reparative or protective environment at later time points.

### Muller cell populations are altered by IL-27

**Fig. 6:**
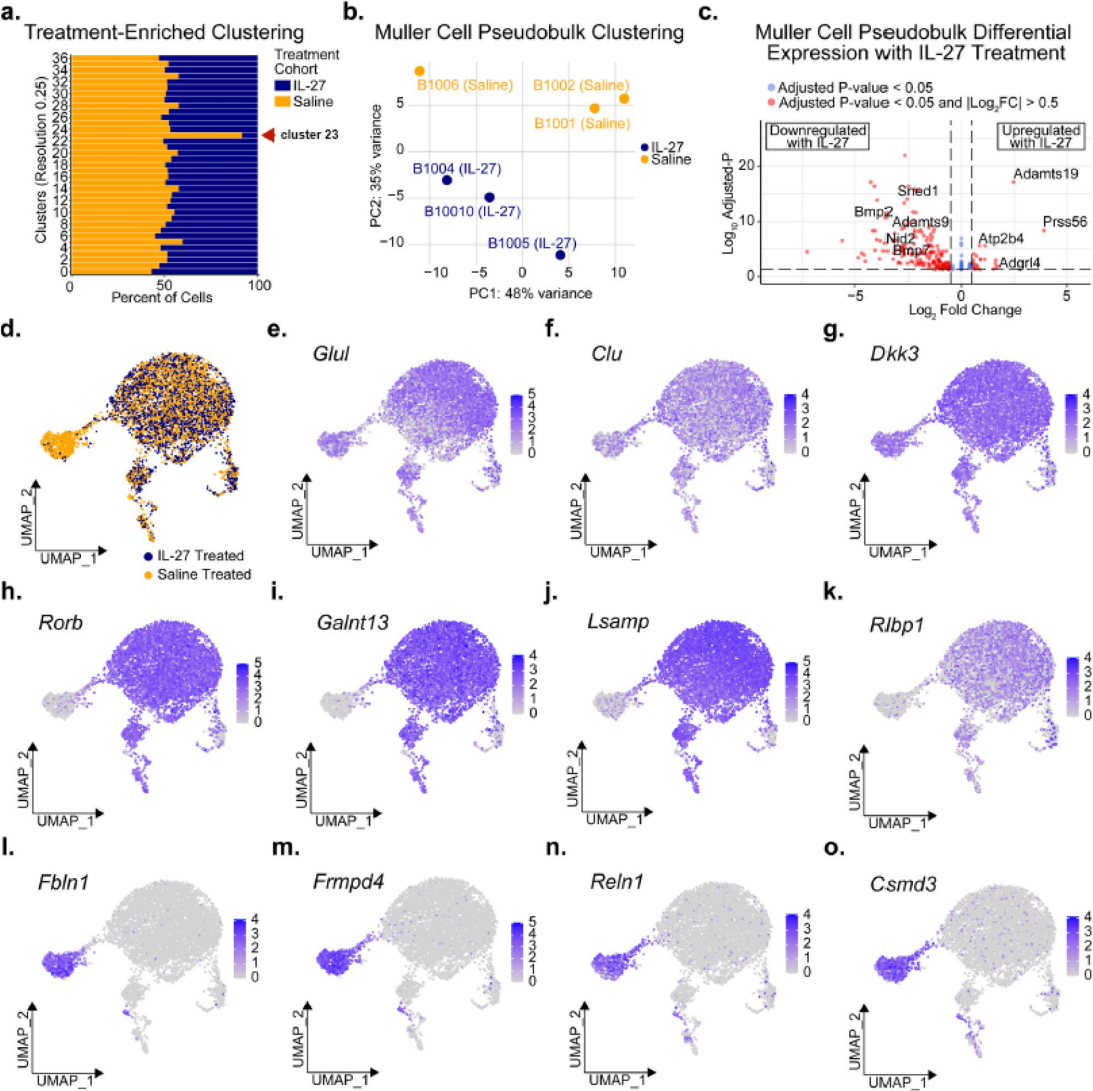
Untreated Muller cells demonstrate increased dysfunction of neurogenesis and extracellular matrix factors compared to IL-27 treated Muller cells. (**a**) Stacked area plot exhibiting the composition of cells within unsupervised Louvain clusters according to treatment group. The red arrow indicates cluster 23, which was enriched in the saline control group (designated in the text as the SIIL subpopulation). (**b**) Principal component analysis (PCA) plot of sum-aggregated pseudobulk samples of Muller cells, calculated from the top 500 most variable genes. (**c**) Differentially expressed genes (DEGs) from pseudobulk analysis of Muller cells, exhibited by volcano plot with y-axis indicating the inverse of the adjusted p-value and with x-axis indicating the log2 fold change (Log2FC). *P*-values and Log2FC was calculated with DESeq2 from pseudobulk counts. *P*-value adjustment calculated with Benjamini-Hochberg procedure. (**d**) UMAP plot of Muller cells (n=5,926 nuclei) annotated by whether nuclei originated from murine retina treated with IL-27 or saline control injections. (**e-o**) UMAP plots demonstrating the normalized RNA expression of (**e-g**) Muller cell makers and (**h-o**) cluster-specific markers

Further assessment of retinal samples revealed at least one saline-enriched cluster (cluster 23), corresponding to a cluster of Muller glia (Fig. 6a) among 36 unique retinal cell clusters identified. Clustering of Muller cell pseudobulk samples by principal component analysis (PCA) further highlighted strong treatment-associated transcriptomic changes within Muller cell populations (Fig. 6b). Pseudobulk differential expression analysis of Muller cell samples from IL-27 treated versus saline control treated retinas revealed 34 significantly upregulated and 212 downregulated differentially expressed genes (DEGs, adjusted p-value < 0.05) (Fig. 6c, Supplementary Data 1). Some of the most significantly upregulated pseudobulk DEGs for Muller cells from IL-27-treated eyes included genes encoding the secreted serine proteases, *Prss56* (log_2_FC = 3.92, adjusted p-value = 7.98 × 10^− 12^) and metalloproteinase *Adamts19* (log_2_FC = 2.47, adjusted p-value = 1.50 × 10^− 21^) involved in regulation of ocular growth (Fig. 6c) [34, 35]. IL-27-treated Muller cells demonstrated transcriptional changes promoting retinal repair, including upregulation of genes involved in regulating Wnt signaling, *Ror2* (log_2_FC = 0.89, adjusted p-value = 7.28 × 10^− 4^) and *Nkd1* (log_2_FC = 1.11, adjusted p-value = 2.64 × 10^− 6^); Wnt signaling functions in Muller glia to protect the retina [36–38]. Additional genes supporting retinal repair associated with IL-27-treated Muller glia included increased retinoid metabolism, with *Rbp1* (log_2_FC = 0.60, adjusted p-value = 0.017) and *Rdh10* upregulation (log_2_FC = 0.57, adjusted p-value = 0.0047), upregulation of neuronal navigator-1, *Nav1* (log_2_FC = 0.56, adjusted p-value = 0.0010), a regulator of cytoskeleton dynamics [39], and significant downregulation of *Bmp2* (log_2_FC=-3.50, adjusted p-value = 2.96 × 10^− 12^), a transcriptional regulator of retinal inflammation that impedes neuronal regeneration and promotes gliogenesis [40, 41]. Furthermore, we found IL-27-treated Muller cells significantly upregulated *Ano1* (log_2_FC = 0.58, adjusted p-value = 7.86 × 10^− 5^), which is involved in aiding neuronal synaptic function [42].

Muller glia from IL-27 treated retinas also demonstrated upregulation of inflammation regulatory genes and neuroprotective-associated factors, including significant upregulation of the neurogenic inflammation and neurovascular regulator *Calcrl* (log_2_FC = 0.69, adjusted *p*-value = 7.41 × 10^− 3^) [43, 44]. Interestingly, we also found increased expression of the neuroinflammatory regulators, *Trim2* (log_2_FC = 0.39, adjusted *p*-value = 0.0038) and *Trim9* (log_2_FC = 0.51, adjusted *p*-value = 0.0035) [45], which are E3 ubiquitin ligases with numerous roles including regulation of Muller cell-derived retinal stem cell differentiation [46]. Other potential inflammatory modulators significantly upregulated with IL27-treated Muller cells included *Sel1l3*, a cellular homeostasis regulator (log_2_FC = 0.50, adjusted *p*-value = 0.040) [47], and neuroinflammation-associated gene *Chn2* (log_2_FC = 0.43, adjusted *p*-value = 0.0078) [48].

Gene set enrichment analysis (GSEA) of pseudobulk-derived DEGs for IL-27 treated Muller glia revealed significantly decreased enrichment of extracellular matrix organization (normalized enrichment score =-1.59, *p*-value = 0.011) due to significant downregulation of genes such as *Sned1*, *Bmp2*, *Bmp7*, *Adamts9* and *Nid2* (Fig. 6c, Supplementary Data 2). Additional interesting pathways included genes in the Nkx2 target group (normalized enrichment score = 1.53, *p*-value = 0.044) (including *Tjp1*,* Nectin3*,* Frmd4b*) that encode adherens junctions, potentially indicating IL-27-treated retinas have increased cell connection integrity.

Muller cell data underwent integration and clustering, reiterating the saline-specific subpopulation (Fig. 6d). There were a total of 7 unique Muller glia clusters and we verified Muller cell marker expression (*Glul*, *Clu*, and *Dkk3*) for all new clusters (Fig. 6e-g). Muller cell clusters shared by nuclei originating from both IL-27 and saline treated retina shared expression of many neuroregulatory factors (Fig. 6h-k). In contrast, the saline-specific subpopulation identified earlier upregulated secreted factors involved in extracellular matrix remodeling, including *Fbln1*, *Fmpd4*, *Reln1* and *Csmd3*, potentially highlighting loss of retina-structural integrity and regulation of glial migration (Fig. 6l-o). Furthermore, genes that were absent from the saline-specific Muller glia subpopulation included genes such as *Rorb*, *Galnt13*, *Lsamp* and *Rlbp1*, involved in retinal function, neurotransmission and metabolism.

To gain further insight into potential roles of the saline-specific Muller glia subpopulation (named SIIL, “*s*uppressed *i*n *IL*-27”, corresponding to cluster 23 in Fig. 6A), we used PANTHER to annotate gene categories that were differentially expressed. GO annotation analysis revealed that genes expressed in the saline-specific subpopulation were significantly enriched for categories including *extracellular matrix protein*, *translational protein* and *microtubule binding motor protein* compared to the other six Muller glia clusters (Table 3). In comparison, gene categories downregulated in saline-specific Muller glia subpopulation included *chemokine*, *Toll-receptor signaling pathway* and *interleukin signaling pathway*. These findings suggest that the saline-specific Muller glia subpopulation lacks immune-related signaling pathways and that IL-27 may shift Muller glia populations toward an immunoresponsive and protective phenotype. Together, we identified a unique cluster of Muller glial cells, which was virtually absent in IL-27 treated animals, enriched for extracellular matrix and cytoskeletal remodeling genes, indicative of a reactive (gliosis-like) phenotype that was markedly reduced following IL-27 treatment.

**Table 3:** Panther GO categories for top differentially expressed genes (increased and decreased) for SIIL Muller glia, which are the saline-specific Muller glia subpopulation that is suppressed by IL-27 treatment

SIIL Subpopulation	GO Category	# Genes	Fold Enrichment	Raw P Value	FDR
Increased	Translational protein	62	2.62	2.24E-12	1.47E-10
	Extracellular matrix protein	33	3.22	1.57E-09	5.16E-08
	Wnt-protein binding	10	5.6	4.38E-06	1.20E-04
	5HT2 type receptor mediated signaling	15	3.26	3.88E-05	3.12E-03
	Growth factor	17	2.6	2.37E-04	3.89E-03
	C4 zinc finger nuclear receptor	11	3.14	5.76E-04	8.73E-03
	Electron transfer activity	4	7.27	1.24E-03	1.50E-02
	Eye morphogenesis	4	7.27	1.24E-03	2.08E-02
	Microtubule binding motor protein	12	2.49	2.74E-03	3.18E-02
	TGF-beta binding	3	7.27	5.54E-03	5.00E-02
Decreased	Chemokine	5	11.2	2.61E-05	4.68E-04
	ATP-binding cassette transporter	13	4.48	3.96E-06	1.30E-04
	CCKR signaling map	24	2.62	1.43E-05	3.29E-04
	Metalloprotease	23	2.5	4.70E-05	7.13E-04
	Lyase	18	2.41	4.77E-04	4.48E-03
	Axon guidance by semaphorins	6	4.89	1.03E-03	1.10E-02
	Interleukin signaling pathway	13	2.48	2.14E-03	1.64E-02
	Toll receptor signaling pathway	9	2.93	3.19E-03	2.23E-02
	HMG box transcription factor	6	3.36	7.76E-03	4.25E-02
	Growth factor receptor binding	8	2.87	6.11E-03	4.45E-02

### IL-27 reshapes retinal cell–cell communication networks toward neurotrophic and anti-inflammatory signaling

**Fig. 7:**
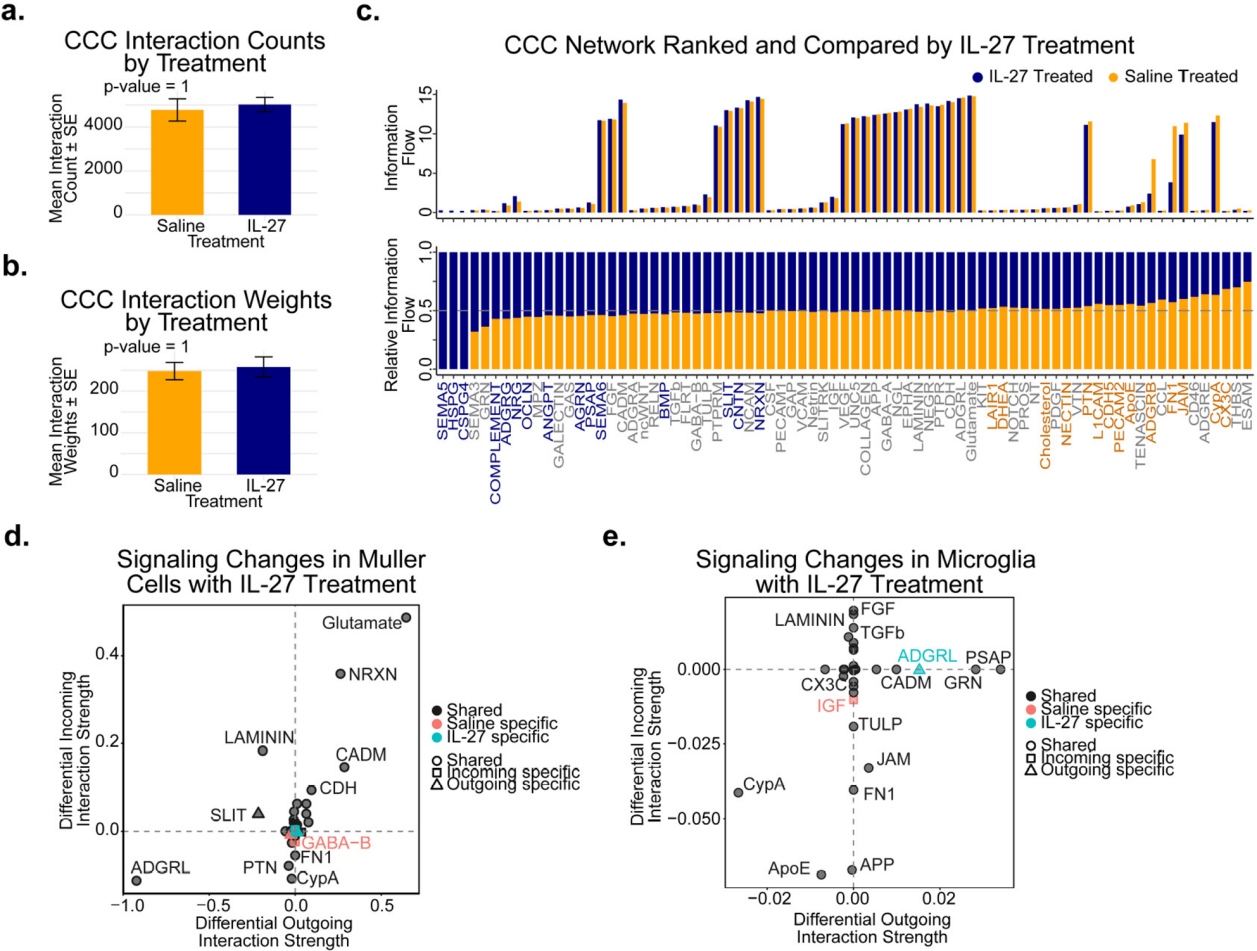
Altered cellular communication within the retinal microenvironment associated with IL-27 treatment. (**a-b**) Bar plots exhibiting the mean cell-cell communication (CCC) (**a**) interaction counts and (**b**) weights calculated for samples from murine retinas treated with IL-27 (n=3) and saline control (n=3) injections. (**c**) Bar plots depicting CCC information flow analysis quantifying the strength of interaction within the CCC network. The paired bar plot (top) compares total probabilities for a communication pathway, and the stacked bar plot (bottom) compares the relative abundance of communication probabilities for a signaling pathway for IL-27 treated versus saline control retinas. Non-significant pathways are labelled in grey, while significant (*p*-value < 0.05) signaling pathways associated with IL-27 and saline groups are labelled in dark blue and orange, respectively. (**d-e**) Differential incoming and outgoing signaling in (**d**) Muller cells and in (**e**) microglia from retinas treated with IL-27 compared to saline control, exhibited by scatter plot of signaling pathways. The x- and y-axes indicate differential outgoing and incoming signaling strengths, respectively. Signaling pathway label color indicates if the pathway is specific to a treatment group, while the shape indicates if the signal is incoming, outgoing, or both. Significance determined by two-tailed Wilcoxon test. Signaling pathway abbreviations collected from the CellChat database

To identify cellular signaling pathways associated with IL-27 treatment within the retina, we quantified cell-cell communication networks utilizing algorithms implemented via CellChat. We observed a non-significant increase in cell-cell communication interaction counts for IL-27 treated (mean count = 5023, median = 5318) versus saline treated (mean count = 4779, median = 5114) retina (Fig. 7a). Similarly, network interaction weights between cells exhibited a non-significant slight increase for IL-27 treated (mean weight = 258.1, median = 274.3) compared to saline treated (mean weight = 248.2, median = 261.8) groups (Fig. 7b). Next, we sought significant cell-cell communication alterations in the retinal microenvironment associated with IL-27 treated compared to saline control samples. Ranked communication network analysis indicated several signaling pathways significantly (p-value < 0.05) associated with IL-27 treatment or saline controls (Fig. 7c). Signaling pathways significantly enriched with and specific to the IL-27 treated microenvironment included Semaphorin 5 A (SEMA5), Heparan Sulfate Proteoglycan (HSPG), and Chondroitin Sulfate Proteoglycan 4 (CSPG4) signaling. SEMA5 and HSPG signaling mediates retinal function, glial migration and neural connectivity [49, 50], and CSPG4 signaling is associated with cell adhesion and glial proliferation and scar formation [51] (Fig. 7c). Other noteworthy signaling pathways significantly enriched in IL-27 treated retina included Neuregulin (NRG), which regulates neuronal synaptic plasticity [52], Neurexin (NRXN), which maintains visual function by regulating retinoid transport and axon-glial interactions [53], and prosaposin (PSAP) signaling, a neurotrophic signaling factor that promotes neuroprotection and axon regeneration [54] (Fig. 7c).

Additional signaling pathways found significantly enriched in saline control retinas compared to IL-27 treated retinas included increased signaling by Adhesion G protein-coupled receptor B (ADGRB), Fibronectin 1 (FN1), and Junctional Adhesion Molecules (JAM) (Fig. 7c), suggesting altered matrix assembly and adhesion signaling within retinas of the saline control cohort [55–57]. Additionally, saline treated retinas demonstrated significantly increased Cyclophilin A (CypA) and Fractalkine (CX3C) signaling (Fig. 7c), both of which are involved in neuroinflammation response [58, 59]. Consistent with the identified IL-27 treated and saline control associated signaling pathways, Muller glia from IL-27 treated cohort demonstrated increased NRXN and decreased FN1 signaling received from cells within the retinal microenvironment (Fig. 7d). Interestingly, Muller glia from IL-27 treated retinas received increased glutamate signaling while Muller glia from saline control retinas had received significantly more GABA-B signaling (Fig. 7d), suggesting altered synaptic signaling is associated with IL-27 treatment.

Given that altered neuroinflammatory signaling pathways were associated with IL-27, we next analyzed differential signaling to and from microglia within the retinal microenvironment (Fig. 7e). Strikingly, one of the most increased outgoing signaling pathways in microglia from IL-27 treated retinas was the neurotrophic signaling factor PSAP that promotes microglial polarization to an anti-inflammatory M2 phenotype when extracellular and promotes a pro-inflammatory M1 phenotype when intracellular [60, 61] (Fig. 7e). Simultaneously, IL-27 treated microglia exhibited decreased neuroinflammation-associated CypA signaling (Fig. 7e). These findings are consistent with reduced cytokine levels at the later timepoints and provide further evidence that IL-27 modifies the retinal inflammatory environment. Together, these findings indicate that IL-27 treatment reorganizes the retinal cell-cell communication network, enhancing neurotrophic and synaptic signaling while suppressing pro-inflammatory and adhesion-driven pathways, thereby promoting a neuroprotective retinal microenvironment.

### IHC analysis of genes differentially expressed in Muller glia subpopulations

**Fig. 8:**
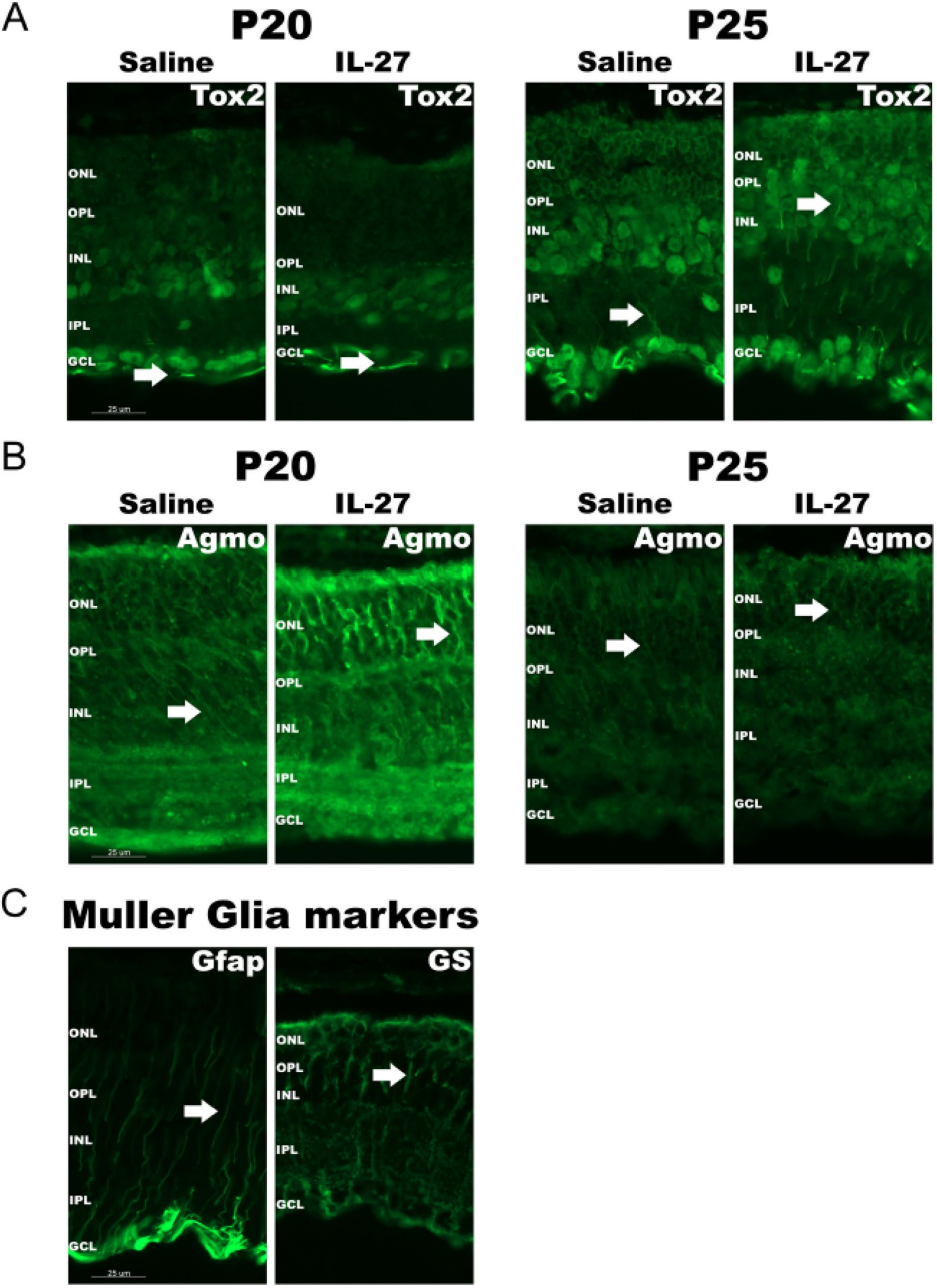
Immunodetection of Tox2 and Agmo. (**A**) Representative IHC images of retinal cryosections showing Tox2 expression at P20 and P25 in saline- and IL-27-treated rd10 eyes. Higher immunodetection of Tox2 in a pattern consistent with Muller glia radial processes (arrow) is observed in IL-27 treated retinas compared with control at P25. (**B**) Representative IHC images of retinal cryosections showing Agmo expression at P20 and P25 in saline- and IL-27-treated rd10 eyes. Intense immunodetection was shown at P20 in both treatments with minimal staining at P25. Detection of Agmo in a pattern consistent with Muller glial radial processes is indicated by the arrows. (**C**) Immunodetection of Muller glia markers GFAP and glutamine synthetase (GS) to demonstrate the morphologies of the glial radial processes (arrows). Retinal layers are indicated as follows: ONL, outer nuclear layer, OPL, outer plexiform layer, IPL, inner plexiform layer, INL, inner nuclear layer, GCL, ganglion cell layer. Scale bars, 25 µm

We next used IHC to assess the expression pattern of genes that were present or absent in the IL-27-suppressed SIIL Muller glia subpopulation. We analyzed genes that were enriched in the IL-27-suppressed SIIL Muller glia subpopulation (Fbln1, Frmpd4, Nox4) and genes that were absent from the subpopulation (Lsamp, Nostrin, Dio2, Glis1, Agmo, Ninj2, Rorb, Tox2 and Pou3f2) (Supplementary Tables 2–3) using their respective antibodies. These genes were selected for analysis because they exhibited large fold changes and represented a wide array of gene classes, including transcription factors, metabolic enzymes, scaffolding proteins and cell adhesion proteins. Immunodetection was performed on retinas from mice at two days (P20, early in disease) or 1 week (P25, peak of rod death) after injection of IL-27 or saline. Several proteins showed different immunodetection patterns in IL-27 injected eyes compared to saline (Fig. 8, negative controls shown in Supplemental Fig. 4), suggesting that they may play a role in the IL-27 response. For example, the transcription factor Tox2 (Fig. 8A), which regulates immune responses in T and NK cells [62], showed more immunostaining within putative Muller glia radial processes in IL-27 treated retinas compared with control at P25. Immunolabeling with the Muller glia markers Gfap and glutamine synthetase (GS), which detect various radial processes morphologies, is shown for comparison (Fig. 8C). The Tox2-positive radial processes were present at P20 but less prominent than at P25, and Tox2 was also localized to lateral striations within the GCL in both IL-27 and saline groups (Fig. 8). Tox2 was also detected within nuclei in the GCL and INL (Fig. 8A). We also investigated the localization of Agmo (alkylglycerol monooxygenase), which is an enzyme that cleaves ether lipids and regulates Wnt signaling [63]. Agmo was detected in Muller glia-like radial processes in both IL-27 and saline treatments at P20, and showed a similar but weaker immunodetection pattern at P25 (Fig. 8B). However, these IHC did not demonstrate differential expression in Muller glia subpopulations, likely because the SIIL subpopulation represented a very small population of Muller glia that could not be distinguished in retinal cross-sections.

Nox4 was analyzed next because it was more highly expressed in the saline-specific Muller glia SIIL subpopulation compared to the other subpopulations. Nox4 induces reactive oxygen species in glia and promotes oxidative stress, inflammation and degeneration. Nox4 immunodetection was localized to the IPL, OPL and IS/OS in both IL-27 and saline injected mice at P20 and P25 (Supplemental Fig. 2). The other proteins that were examined, Dio2, Fbln1, Frmpd4, Glis1, Lsamp, Ninj2, Nostrin, Pou3f2 and Rorb, showed varying detection patterns, including in putative Muller glia radial processes, INL and other layers (Supplemental Fig. 3). Similar to Tox2 and Agmo, these proteins did not show differences in localization between treatments despite the snRNA-seq analysis demonstrating expression differences in the saline enriched subpopulation. These findings indicate that IL-27 modifies protein expression and localization within Muller glia, and future studies using RNAscope or flow cytometry will further investigate selective suppression of genes in the SIIL cluster 23 subpopulation.

## Discussion

In this study, we tested the hypothesis that reducing inflammation at early stages of retinal disease, prior to substantial photoreceptor loss, could prolong vision rescue. We demonstrated that IL-27 delivery to rd10 mice decreased inflammatory cytokines and induced sustained retina protection for at least four weeks after injection. Additionally, gene expression analysis two days after injection demonstrated that IL-27 led to suppression of a unique Muller glia subpopulation that expressed genes involved in cellular adhesion and extracellular matrix while lacking expression of inflammatory genes. These findings demonstrate that reducing reactive inflammation early in the pathology of retinal degeneration using IL-27 is sufficient for neuroprotection in rd10 mice for at least one month. Additionally, our results suggest that suppression of a reactive Muller glia subpopulation and regulation of critical glial signaling pathways contribute to the neuroprotective effect of IL-27.

A major goal of this study was to identify molecular and cellular mechanisms by which IL-27 induces photoreceptor survival. Consistent with the known function of IL-27 as an anti-inflammatory cytokine, we observed reduced pro-inflammatory cytokines and chemokines in retinas a week after IL-27 injection and in a microglial cell line treated with IL-27. The snRNA-seq analysis also indicated reduced inflammatory signaling in Muller glia and microglia. Although several anti-inflammatory cytokines were decreased in Il-27 injected eyes, the overall effect of IL-27 was anti-inflammatory and neuroprotective. Furthermore, CellChat analyses of the snRNA-seq data indicated several signaling pathways are altered by IL-27 in Muller glia, including multiple inflammatory pathways, and regulators of cellular migration, adhesion and extracellular matrix, suggesting potential mechanisms by which Muller glia contribute to the protective effect of IL-27. Therefore, our data suggests that reducing inflammatory pathways early in degeneration by delivering IL-27 reshapes the retinal microenvironment, establishing conditions that reduce inflammatory responses later in disease when photoreceptor death and inflammation are most pronounced.

Another potential mechanism of IL-27-mediated protection was suggested by previous studies showing IL-27 reduced apoptosis by inducing anti-apoptotic genes directly in neurons and other cell types [16, 64]. Although photoreceptors in rd10 mice show mild structural changes at the early timepoint assessed by snRNA-seq (P20), excessive cell death in rd10 mice is not observed until P25 and later [5, 33]. However, our snRNA-seq analysis detected differential expression of numerous cell survival genes, and the GO categories *regulation of apoptotic process* and *regulation of necroptotic cell death* were enriched in down-regulated genes in cone and rod photoreceptors. This finding suggests that during early pathologic changes, IL-27 regulates expression of cell survival genes that may contribute to a reparative and protective environment. Heat shock proteins hsp70 and hsp90 were also decreased in IL-27 injected eyes, indicating lower cell stress. Activation of inflammatory pathways during photoreceptor degeneration, for example by secretion of TNFα and other cytokines and oxidation of macromolecules, directly induces apoptosis and necroptosis [65]. IL-27 may directly inhibit cell death pathways or may indirectly block them by suppressing inflammation. Therefore, IL-27-induced suppression of cell death genes provides further evidence that IL-27 promotes establishment of a protective microenvironment that leads to photoreceptor protection when measured at least four weeks later. Future work will investigate how the differentially expressed cell death genes identified here contribute to IL-27-mediated photoreceptor protection.

Another interesting finding was that reduced translation and ATP synthesis/oxidative phosphorylation genes were observed in several cell types, including microglia and Muller glia. These results suggest that lower metabolic demand is associated with reduced inflammation and lower glial reactivity. Similarly, reduced metabolism and translation genes were also reported in snRNAseq analysis of Muller glia from AMD retinas [66]. Future studies will determine whether IL-27 directly or indirectly regulates mitochondria function and will further characterize the relationship between energy metabolism and IL-27-mediated retina protection.

A novel finding in this study was the disappearance of a Muller glia subpopulation that we named SIIL (which appears as the saline-specific Muller glia cluster 23 in Fig. 6) in IL-27 treated retinas early in degeneration. A potential role of this saline-specific/IL-27 suppressed Muller glia subpopulation is suggested by the finding that it has lower expression of inflammatory regulating genes, such as TLRs and chemokines, compared to other Muller glia. The SIIL Muller glia subpopulation also showed down-regulation *of GFAP*,* Cralbp*,* Apoe* and *Stat* transcription factor genes, suggesting that they are a non-responsive and potentially non-protective population of Muller glia. Upregulated genes in the saline-specific Muller glia subpopulation include growth factors, adhesion proteins and extracellular matrix structural proteins, suggesting that these Muller glia may contribute to retinal remodeling and glial scarring during degeneration. Therefore, the SIIL subpopulation may represent Muller glia that do not mediate or contribute to immune signaling and are reduced when IL-27 promotes an anti-inflammatory environment. Suppression of the SIIL Muller glia subpopulation by IL-27 is consistent with neuroprotection and reduced remodeling in retinas that have lower photoreceptor death. Therefore, these findings suggest that IL-27 shifts Muller glia to a more damage-responsive phenotype, which may contribute to sustained photoreceptor protection. Future studies will determine whether these glia cells contribute to degeneration and whether their absence is required for IL-27-mediated protection.

Muller glia are heterogeneous, and subpopulations have been reported based on varying gene expression, function and location in mouse, zebrafish and human retinas, especially during retinal stress [67]. However, the significance of Muller glia heterogeneity to disease pathogenesis or intrinsic tissue repair is unknown. Several studies suggest that subpopulations of Muller glia may be responsible for responding to retinal damage. For example, in light-damaged zebrafish retinas, three Muller glia populations were identified that differed in proliferation capability and expression of Stat3 and other regeneration promoting genes [68]. Similarly, at least four Muller glia subpopulations that contribute to neuronal regeneration and glial replenishment were identified using scRNA-seq in damaged zebrafish retinas [69]. In the light-induced retinal damage model in mice, a Muller glia subpopulation that expressed growth factor and synaptogenesis related genes was identified 2 days after injury, suggesting a role in retinal repair [70]. An analysis of retinas from AMD patients [71] demonstrated three Muller glia subpopulations based on expression of key marker genes, and the NaIO3-induced AMD mouse model showed six Muller glia subpopulations [72]. However, comparison of the marker genes for the Muller glia subpopulations in Aredo et al. and Menon et al. studies to the seven clusters identified in the current study did not show similar differential expression patterns, suggesting distinct Muller glia subpopulations were present in the rd10 retinas.

To our knowledge, our study provides the first identification of Muller glia subpopulations that change in association with a neuroprotective treatment and highlights the dynamic nature of Muller glia heterogeneity and function under disease and treatment conditions. Given the essential roles of Muller glia in photoreceptor survival and retinal homeostasis, characterizing these heterogeneous Muller glia responses will be crucial to determining underlying mechanisms by which Muller glia protect, or fail to protect, retinal neurons. Future studies will characterize the dynamic properties of the different Muller glia subpopulations, their interactions with each other, with microglia and other inflammatory cells, and their functional roles during treatment. Additionally, IHC analysis on selected proteins differentially expressed in Muller glia subpopulations revealed intriguing results. For example, the immune transcription factor Tox2 and metabolic enzyme Agmo showed localization in Muller glia-like processes. Therefore, these proteins may play roles in Muller glia and are potentially involved in photoreceptor protection. Although IHC is semi-quantitative, there were differences in immunodetection of these proteins and others between treatments and timepoints. Future work will investigate the contribution of these proteins to IL-27 function. However, we did not detect localization of any of the proteins tested to a subset of Muller glia in the retina sections, which was likely due to the SIIL subpopulation representing a small number of cells in the retina, or that post-transcriptional regulation reduced expression differences among subpopulations.

## Conclusions

In conclusion, this study demonstrated that an early single intravitreal injection of recombinant IL-27 led to sustained photoreceptor survival and vision rescue at least four weeks after injection. IL-27 altered inflammatory pathways during retinal degeneration and suppressed genes involved in apoptosis, protein translation and metabolism, indicating complex multi-pathway mechanisms involved in IL-27-mediated neuroprotection. These findings predict that additional IL-27 injections or sustained delivery would continue to suppress damaging inflammation and lead to long-term photoreceptor survival. Furthermore, we demonstrated the novel finding that IL-27 resulted in suppression of a Muller glia subpopulation that expressed genes that mediate cellular adhesion and extracellular matrix and that have lower expression of inflammatory genes. Therefore, these findings suggest several mechanisms by which IL-27 functions to restore homeostasis and dampen retinal inflammation early in degeneration to mediate neuroprotection later in degeneration.

## Data Availability

All snRNA-seq gene expression data is deposited in the Gene Expression Omnibus (GEO), accession number GSE307302. Additional data generated in the current study are available from the corresponding author on reasonable request.

## Supplementary nformation

Supplementary Material 1. Supplemental Table 1: Primers used in the study.

Supplementary Material 2. Supplemental Table 2: Differential expression of genes from the SIIL Muller glia subpopulation that were examined by IHC.

Supplementary Material 3. Supplemental Table 3: Source data for CellChat pathway information flow analysis included in Figure 7c, generated with the function “rankNet.” The table includes aggregated, scaled, and relative pathway contributions by treatment group (Saline or IL27), sorted by p-values calculated with a two-tailed Wilcoxon test.

Supplementary Material 4. Supplemental Figure 1: Detailed overview of single-nucleus annotation of IL-27 murine retina with cell cycle analysis. (a-b) Uniform Manifold Approximation and Projection (UMAP) dimension reduction plot displaying 51,988 single nuclei annotated according to (a) unsupervised Louvain clusters (determined with resolution equal to 0.25) and to (b) detailed cell subtypes from reference-based mapping. (c) Stacked area plot exhibiting the composition of cells within unsupervised Louvain clusters according to major cell type.

Supplementary Material 5. Supplemental Figure 2. Immunodetection of Nox4. Representative IHC images of retinal cryosections showing Nox4 expression at P20 and P25 in saline- and IL-27 treated rd10 eyes. Retinal layers are indicated as follows: ONL, outer nuclear layer, OPL, outer plexiform layer, IPL, inner plexiform layer, INL, inner nuclear layer, GCL, ganglion cell layer. Scale bars, 25 µm.

Supplementary Material 6. Supplemental Figure 3. Representative IHC images of genes differentially expressed in the SIIL Muller glia subpopulation. Retinal cryosections were immunostained with antibodies against Dio2, Fbln1, Frmpd4, Glis1, Lsamp, Ninj2, Nostrin, Pou3f2 and Rorb. All saline and IL-27 images for a given gene were acquired at identical exposures; saline and IL-27 images were equivalently brightened for select genes (Fbln1, Frmpd4, Glis1, Lsamp, Ninj2 and Rorb) to enhance visualization of immunostaining patterns. Left labels indicate retinal layers: ONL = outer nuclear layer, OPL = outer plexiform layer, IPL = inner plexiform layer, INL = inner nuclear layer, GCL = ganglion cell layer. Scale bars = 25 µm.

Supplementary Material 7. Supplemental Figure 4. Negative control for IHC for a retina section from P20 demonstrating no background immunodetection with the secondary antibody.

## Abbreviations

IL-27: Interleukin-27
snRNA-seq: single nucleus RNA sequencing
ONL: Outer nuclear layer
OPL: Outer plexiform layer
IPL: Inner plexiform layer
INL: Inner nuclear layer
GCL: Ganglion cell layer
IRD: Inherited retinal diseases
P: Post-natal day
OMR: Optomotor response tracking,
ERG: Electroretinogram
GO: Gene ontology
