# Supplementary material for: Interleukin 27-induced photoreceptor survival is associated with suppression of a novel Muller glia subpopulation
Source: Cell Commun Signal. 2026 Apr 16;24:302. doi: 10.1186/s12964-026-02885-1 (PMC13200363; doi:10.1186/s12964-026-02885-1)
Supplement: Supplementary file 1 — Supplementary Material 1. Supplemental Table 1: Primers used in the study. [file 12964_2026_2885_MOESM1_ESM.pdf]

Table 1. Primers used in this study.

Gene Name	Direction	Sequence 5'−3'
Arg1	Forward	CTCCAAGCCAAAGTCCTTAGAG
Arg1	Reverse	AGGAGCTGTCATTAGGGACATC
Gal3	Forward	GTCCGGAGCCAGCCAACGAG
Gal3	Reverse	CTGGTTCCCCCATGCGCCAG
IL-1α	Forward	GTCAACTCATTGGCGCTTGA
IL-1α	Reverse	CGTTGCTTGACGTTGCTGAT
NOS2	Forward	CAGAGGACCCAGAGACAAGC
NOS2	Reverse	TGCTGAAACATTTCCTGTGC
P2RY12	Forward	TACCCTACAGAAACACTCAAGG
P2RY12	Reverse	AAGGTGGTATTGGCTGAGGTG
TGFβ	Forward	ACTGGAGTTGTACGGCAGTG
TGFβ	Reverse	GGGGCTGATCCCGTTGATTT
TNFα	Forward	CTGATGAGAGGGAGGCCATT
TNFα	Reverse	AGCACAGAAAGCATGATCCG
TREM2	Forward	CAGCCCTGTCCCAAGCCCTCAAC
TREM2	Reverse	CTCCTCACCCAGCTGCCGACACC
