# Supplementary material for: Interleukin 27-induced photoreceptor survival is associated with suppression of a novel Muller glia subpopulation
Source: Cell Commun Signal. 2026 Apr 16;24:302. doi: 10.1186/s12964-026-02885-1 (PMC13200363; doi:10.1186/s12964-026-02885-1)
Supplement: Supplementary file 2 — Supplementary Material 2. Supplemental Table 2: Differential expression of genes from the SIIL Muller glia subpopulation that were examined by IHC. [file 12964_2026_2885_MOESM2_ESM.pdf]

Gene ID	Gene Name	SIIL Subpopulation		Saline vs. IL-27		Protein Class
		logFC	adjusted Pvalue	logFC	FDR	
Fbln1	Fibulin-1	7.901866707	0	2.7606699	7.42E-215	Cell Adhesion Molecule / ECM
Lsamp	Limbic system-associated membrane protein	-3.469653981	7.3225E-285	-0.13040807	0.00018092	Cell Adhesion Molecule
Ninj2	Ninjurin-2	-3.623834561	7.58092E-11	-0.1123791	0.157538033	Cell Adhesion Molecule
Agmo	Alkylglycerol monooxygenase	-4.652132931	5.88122E-23	-0.158747108	0.033344131	Metabolic enzyme
Dio2	Type II iodothyronine deiodinase	-5.064426121	1.46E-14	-0.046849801	0.622492407	Metabolic enzyme
Nox4	NADPH oxidase 4	5.641068879	1.59E-89	nd	nd	Metabolic enzyme
Frmpd4	FERM and PDZ domain-containing protein 4	7.966895548	0	3.487345494	0	Scaffolding (adaptor) Protein
Nostrin	Nostrin	-5.645198632	1.27326E-12	0.050099	0.57106681	Scaffolding (adaptor) Protein
Glis1	Zinc finger protein GLIS1	-4.92971773	3.79044E-05	-0.114168896	0.111976406	Transcription Factor
Pou3f2	POU domain, class 3, transcription factor 2	-5.322668099	0.000646988	nd	nd	Transcription Factor
Rorb	Nuclear receptor ROR-beta	-3.657054006	1.7008E-294	-0.21572264	2.69372E-12	Transcription Factor
Tox2	TOX high mobility group box family member 2	-4.056905925	9.1655E-126	-0.156880615	0.000617996	Transcription Factor
