# Supplementary figures and images for: Interleukin 27-induced photoreceptor survival is associated with suppression of a novel Muller glia subpopulation
Source: Cell Commun Signal. 2026 Apr 16;24:302. doi: 10.1186/s12964-026-02885-1 (PMC13200363; doi:10.1186/s12964-026-02885-1)

a.

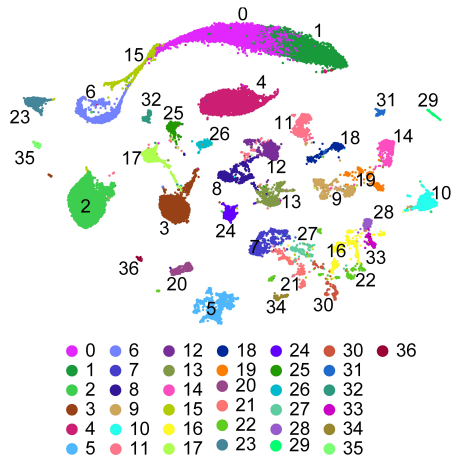

b.

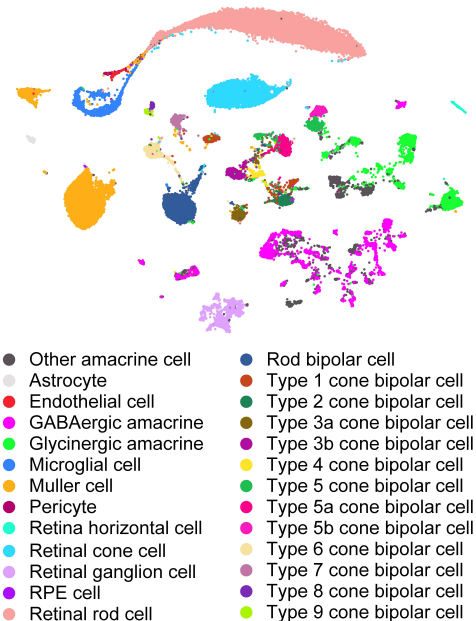

c.

## Cell Type Composition of Clusters

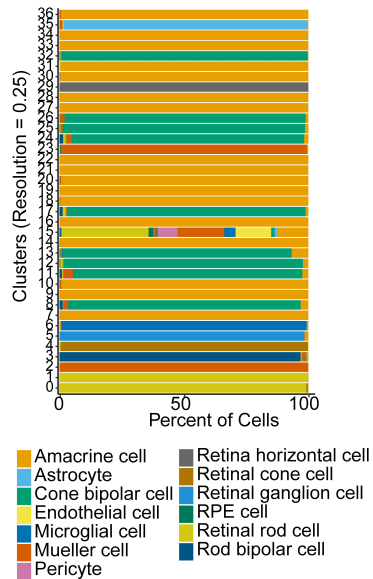

Suppl Fig1

Supplement: Supplementary file 4 — Supplementary Material 4. Supplemental Figure 1: Detailed overview of single-nucleus annotation of IL-27 murine retina with cell cycle analysis. (a-b) Uniform Manifold Approximation and Projection (UMAP) dimension reduction plot displaying 51,988 single nuclei annotated according to (a) unsupervised Louvain clusters (determined with resolution equal to 0.25) and to (b) detailed cell subtypes from reference-based mapping. (c) Stacked area plot exhibiting the composition of cells within unsupervised Louvain clusters according to major cell type. [file 12964_2026_2885_MOESM4_ESM.pdf]

**P20**

**Saline**

**IL-27**

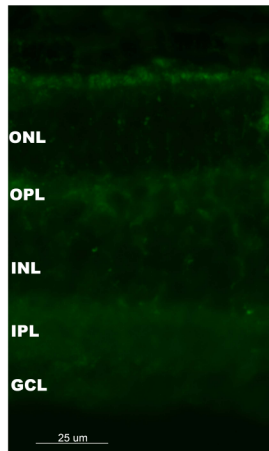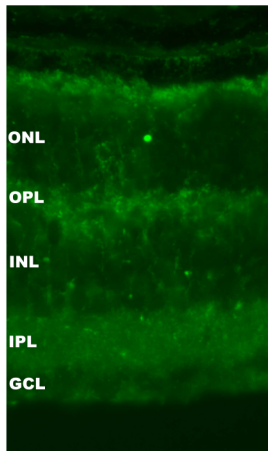

**P25**

**Saline**

**IL-27**

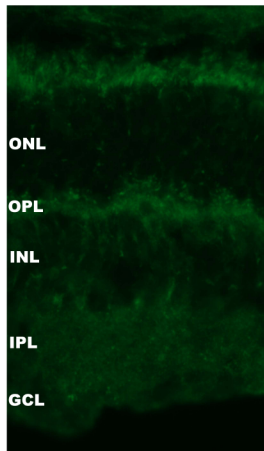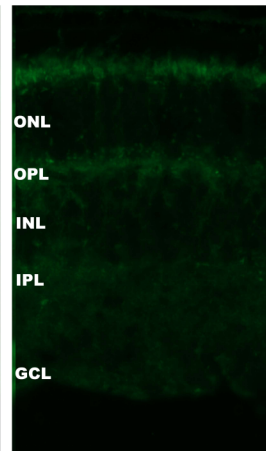

Supplement: Supplementary file 5 — Supplementary Material 5. Supplemental Figure 2. Immunodetection of Nox4. Representative IHC images of retinal cryosections showing Nox4 expression at P20 and P25 in saline- and IL-27 treated rd10 eyes. Retinal layers are indicated as follows: ONL, outer nuclear layer, OPL, outer plexiform layer, IPL, inner plexiform layer, INL, inner nuclear layer, GCL, ganglion cell layer. Scale bars, 25 µm. [file 12964_2026_2885_MOESM5_ESM.pdf]

SF2

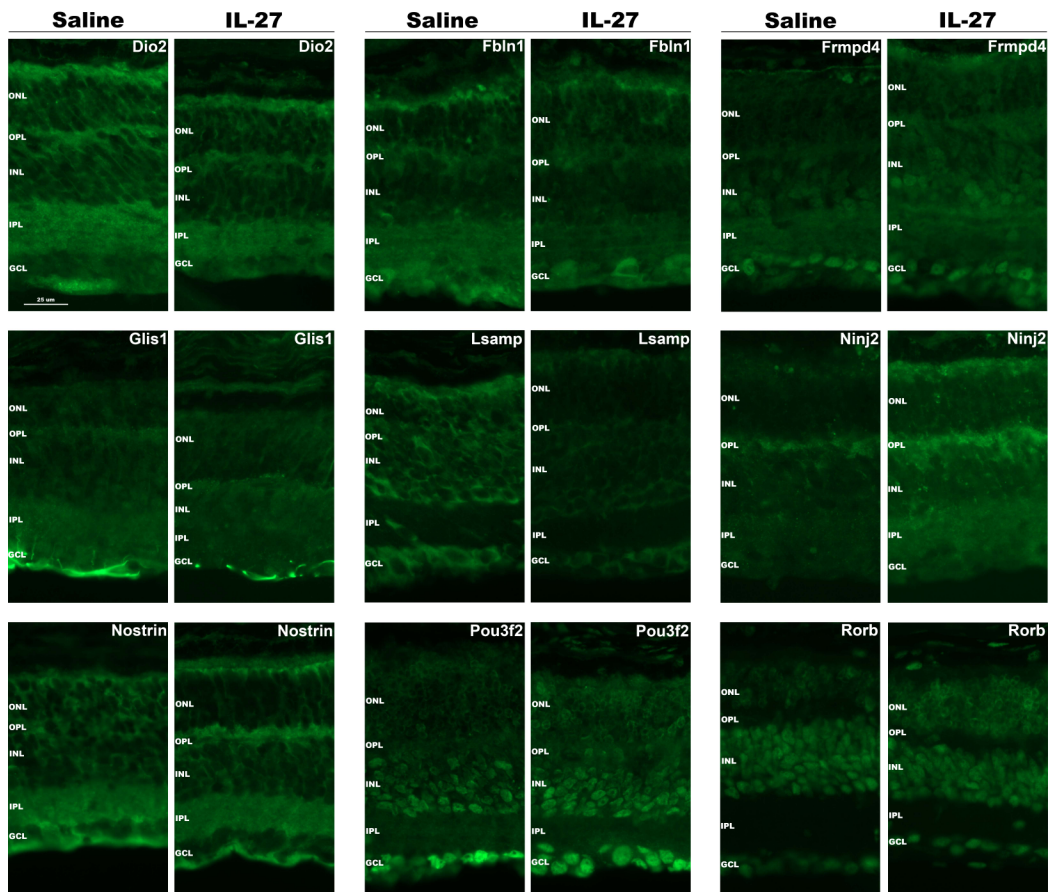

Supplement: Supplementary file 6 — Supplementary Material 6. Supplemental Figure 3. Representative IHC images of genes differentially expressed in the SIIL Muller glia subpopulation. Retinal cryosections were immunostained with antibodies against Dio2, Fbln1, Frmpd4, Glis1, Lsamp, Ninj2, Nostrin, Pou3f2 and Rorb. All saline and IL-27 images for a given gene were acquired at identical exposures; saline and IL-27 images were equivalently brightened for select genes (Fbln1, Frmpd4, Glis1, Lsamp, Ninj2 and Rorb) to enhance visualization of immunostaining patterns. Left labels indicate retinal layers: ONL = outer nuclear layer, OPL = outer plexiform layer, IPL = inner plexiform layer, INL = inner nuclear layer, GCL = ganglion cell layer. Scale bars = 25 µm. [file 12964_2026_2885_MOESM6_ESM.pdf]

## Saline Negative

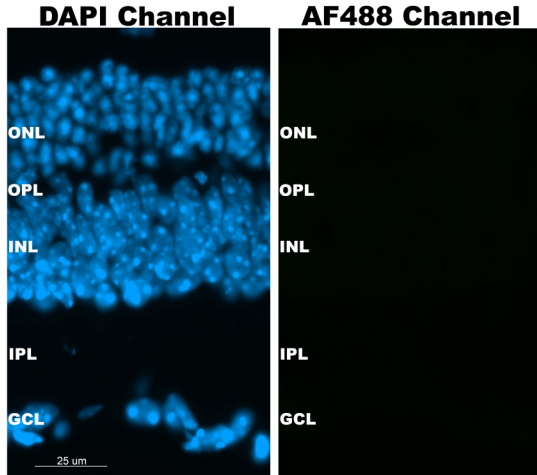

## IL-27 Negative

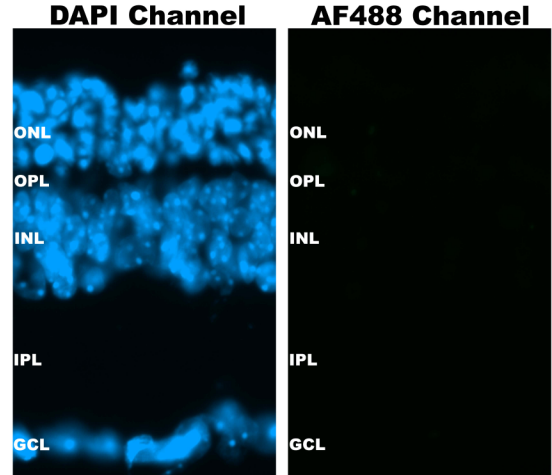

SF3

Supplement: Supplementary file 7 — Supplementary Material 7. Supplemental Figure 4. Negative control for IHC for a retina section from P20 demonstrating no background immunodetection with the secondary antibody. [file 12964_2026_2885_MOESM7_ESM.pdf]
